# Decision aid development and its acceptability among adults with attention‐deficit/hyperactivity disorders regarding treatment discontinuation after remission

**DOI:** 10.1002/pcn5.57

**Published:** 2022-11-14

**Authors:** Noa Tsujii, Takashi Okada, Masahide Usami, Hidenori Kuwabara, Junichi Fujita, Hideki Negoro, Junzo Iida, Yumi Aoki, Yoshikazu Takaesu, Takuya Saito

**Affiliations:** ^1^ Department of Child Mental Health and Development Toyama University Hospital Toyama Toyama Japan; ^2^ Department of Neuropsychiatry Kindai University Faculty of Medicine Osakasayama Osaka Japan; ^3^ Department of Developmental Disorders, National Institute of Mental Health National Center of Neurology and Psychiatry Kodaira Tokyo Japan; ^4^ Department of Child and Adolescent Psychiatry, Kohnodai Hospital National Center for Global Health and Medicine Ichikawa Chiba Japan; ^5^ Senogawa Hospital Hiroshima‐shi Hiroshima Japan; ^6^ Department of Child Psychiatry Yokohama City University Hospital Yokohama Kanagawa Japan; ^7^ Shigisan Hospital Herarland Shigisan Ikoma Nara Japan; ^8^ Medical Corporation Nanfukai Manyo Clinic Child Mental Health Care Center Kizuna Kashihara Nara Japan; ^9^ Graduate School of Nursing St. Luke's International University Chuo‐ku Tokyo Japan; ^10^ Department of Neuropsychiatry, Graduate School of Medicine University of the Ryukyus Okinawa Japan; ^11^ Department of Child and Adolescent Psychiatry, Faculty of Medicine Hokkaido University Sapporo Hokkaido Japan

**Keywords:** attention‐deficit/hyperactivity disorder, decision aid, shared decision making

## Abstract

**Aim:**

Current clinical guidelines for attention‐deficit/hyperactivity disorder (ADHD) put shared decision making (SDM) at the center of care. However, there remain challenges in SDM in ADHD management, particularly regarding the decision to continue or discontinue medication after ADHD remission in adult patients. We aimed to develop a decision aid (DA) for adult patients with ADHD regarding the continuation or discontinuation of their ongoing ADHD medications after they have attained remission.

**Method:**

We systematically developed a DA according to the International Patient Decision Aid Standard (IPDAS). First, we created a DA prototype using the results of our previous systematic review and meta‐analysis that identified the consequences of continuing and discontinuing ADHD medications. Second, we administered a mixed‐method questionnaire (alpha acceptability testing) to adult patients with ADHD and healthcare providers to improve the DA prototype and develop it into a final version that is acceptable for clinical settings.

**Results:**

Our DA consisted of ADHD description, the option to continue or discontinue ADHD medications, the advantages and disadvantages of the consequences, as well as value clarification exercises for each option. Patients (*n* = 20) reported that the DA had acceptable language (85%), adequate information (75%), and a well‐balanced presentation (53%). Healthcare providers (*n* = 19) provided favorable feedback. The final DA met all six IPDAS requisite criteria.

**Conclusions:**

Our results could facilitate the SDM process between patients and healthcare providers on the continuation or discontinuation of ADHD medication following remission. Further studies should verify the effects of using the DA during the SDM process among patients across the age spectrum with ADHD and healthcare providers.

## Graphical abstract

Current clinical guidelines for attention‐deficit/hyperactivity disorder (ADHD) put shared decision making (SDM) at the center of care. We developed a decision aid (DA) for adult patients with ADHD that contained two options regarding the decision to continue or discontinue current ADHD medication following remission. We successfully developed a DA for adult patients with ADHD who are considering continuation or discontinuation of ADHD medication after symptom remission. Further studies should be performed to verify the effects of using the DA during the SDM process for children, adolescents, and adults with ADHD.

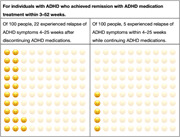

## INTRODUCTION

Attention‐deficit/hyperactivity disorder (ADHD) is a lifespan neurodevelopmental disorder characterized by age‐inappropriate levels of inattention, hyperactivity, and/or impulsivity that impairs daily function and overall quality of life.^1^, ^2^, ^3^ The prevalence rates of this disorder in most cultures are 5.9% and 2.5% for youth and adults, respectively.^4^ The most common treatments for individuals with ADHD are pharmacologic, nonpharmacologic, or a combination of both.^5^

Most clinical guidelines generally recommend that healthcare professionals should involve people receiving care in decision making about screening, treatment, and other interventions to enable informed choices.^6^, ^7^ Shared decision making (SDM) is a joint process in which healthcare professionals working with patients aim to reach a decision about care.^7^ Decision aids (DAs) are evidence‐based intervention tools for facilitating SDM in clinical practice^6^, ^8^ and helping people make informed choices about their healthcare.^7^ A Cochrane review concluded that DAs could improve value‐congruent choices for people receiving a particular intervention compared with usual care across various decision contexts.^8^

Current clinical guidelines for ADHD put SDM at the center of care.^9^, ^10^, ^11^ Several studies have examined the effect of DA on individuals with ADHD or their families. A randomized controlled trial reported that the use of DA resulted in a higher quality of care with respect to ADHD diagnosis, including a prospect for a higher quality of ADHD management in children.^12^ Other studies on ADHD have shown the utility of DAs with regard to ADHD diagnosis,^13^, ^14^ interventions,^13^, ^14^, ^22^ discussion about drug holidays,^15^, ^16^ as well as assessment and management of aggressive and disruptive behavior.^17^

Recently, the prevalence of ADHD medication use has increased among adults worldwide.^18^ Pharmacological treatments for adult patients with ADHD are typically planned for a long term^3^; however, there are concerns about the safety of these treatments.^5^ The potential long‐term risks of pharmacological treatments for adult patients with ADHD have become the centerpiece of most clinical guidelines, with the recommendation that at least an annual review of the treatment regimen is necessary to ascertain the need for the continuation of medications.^10^, ^11^ For instance, the National Institute for Health and Care Excellence (NICE) guideline recommends that healthcare providers encourage people with ADHD to discuss any preferences to stop or change medication and be involved in any decisions about stopping treatments.^11^

Despite the prioritization of SDM for ADHD in most clinical guidelines, there are still challenges against its use during ADHD management, especially regarding the continuation or discontinuation of ADHD medications after remission. Previous studies on the use of DAs for ADHD had focused mainly on children and adolescents with ADHD,^12^, ^15^, ^16^, ^17^ assessment of ADHD,^13^, ^14^, ^16^, ^22^ and initial pharmacological intervention.^13^, ^14^, ^22^ In the present study, we hypothesized that DA is a validated tool for deciding on the continuation or discontinuation of ADHD medications after remission in adult patients. We therefore aimed to develop a DA for adult patients with ADHD regarding the continuation or discontinuation of their ongoing ADHD medications after they have attained remission. In addition, we assessed stakeholders' acceptability of the DA. We assumed that the DA would be used in primary care settings and psychiatric outpatient services.

## METHODS

### Study design

**FIGURE 1:**
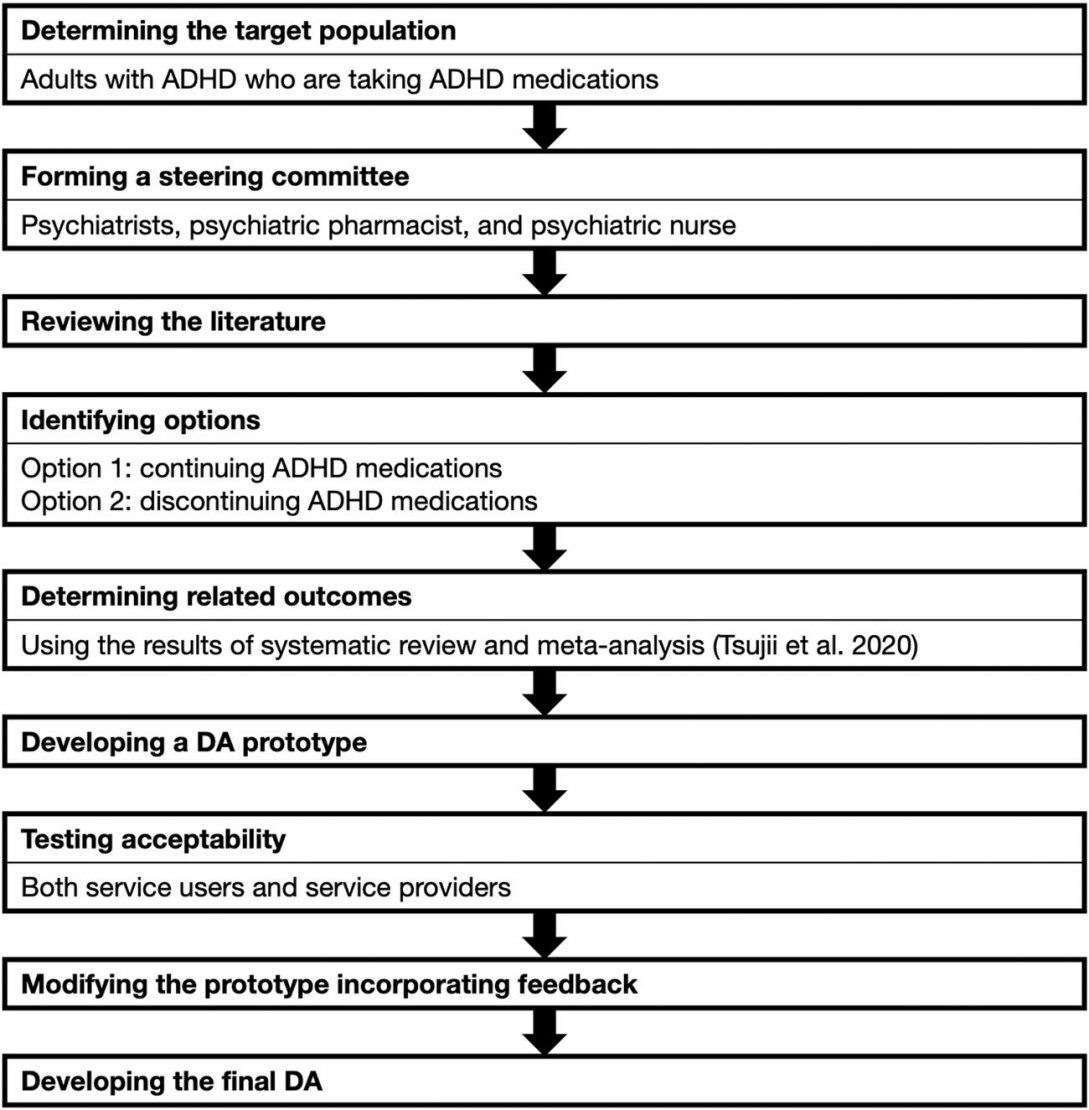
Process of developing a DA for adult patients with ADHD that contained two options for continuation or discontinuation of ADHD medication treatment after remission, following the approach of Coulter et al.^20^ ADHD, attention‐deficit/hyperactivity disorder; DA, decision aid.

We conducted this study in accordance with the Ottawa Decision Support Framework^19^ and International Patient Decision Aid Standards (IPDAS)^20^ (Figure 1). The IPDAS is an evidence‐based framework of criteria established to standardize the content and development process of DAs,^21^ as described previously.^22^ The process includes (1) determining the target population and assessing their decision needs, (2) forming a steering committee of experts, (3) conducting a literature review to determine options and related outcomes, (4) developing a DA prototype, (5) testing the acceptability of the DA prototype among stakeholders, (6) modifying the DA based on the results of the acceptability test to develop a final DA, and (7) field testing of the final version for effectiveness in real clinical settings. This study was approved by the ethics review board of Kyorin University, Tokyo, Japan (approval number R02‐037).

### Target population

We included adult patients with a primary diagnosis of ADHD according to the Diagnostic and Statistical Manual of Mental Disorders, fifth edition (DSM‐5). All study participants provided informed consent. The inclusion criteria were as follows: (1) aged 18 years or older, (2) had been taking at least one ADHD medication, such as methylphenidate, lisdexamfetamine, atomoxetine, or guanfacine, and (3) had experienced remission with ADHD medication treatment. The exclusion criteria were as follows: (1) no response to ADHD medications and no remission experience with the medications, (2) attaining remission in <3 months, and (3) having any other neurodevelopmental disorders, such as autism spectrum disorder, or psychiatric disorders. Healthcare providers who regularly see people with adult ADHD were recruited from the same health facilities used by the patients. The remission and response to medication were defined clinically by each adult ADHD healthcare provider.

### Steering committee

We formed a steering committee that comprised experts on ADHD and DA methodology. The committee comprised six psychiatrists who routinely treated children, adolescents, and adults with ADHD, a psychiatric pharmacist who was routinely involved in psychotropic pharmacotherapy, and a psychiatric nurse who was familiar with SDM literature in psychiatry with adequate experience in developing DAs for ADHD.^13^, ^23^

### Literature review

We conducted a relevant literature review and examined the positive and negative features of the two options: (1) continuing ADHD medications and (2) discontinuing ADHD medications. We also searched literature regarding nonpharmacological approaches for the treatment of ADHD, such as behavioral or psychological interventions.

### Determining the related outcomes

Regarding the related outcomes of the two options, that is, continuing and discontinuing ADHD medications after remission, we used the results of a systematic review and meta‐analysis conducted earlier.^24^ The meta‐analysis findings showed that discontinuing any ADHD medication was significantly associated with the relapse of ADHD symptoms in adult patients with ADHD at a risk ratio (RR) of 3.70.

### Prototype development

We developed a DA prototype in accordance with the IPDAS criteria^21^ using the results of our systematic review and meta‐analysis.^24^

### Acceptability testing

The acceptability test was performed by surveying stakeholders of the DA. A mixed‐methods survey was developed according to the validated acceptability scoring tool, which included the assessment of the comprehensiveness of the DA with regard to its length, amount of information, balance of related information, and ability to assess the targeted decision.^25^ This is the standard process for developing a DA and enabling its improvement for the final version with due consideration of the feedback.

We recruited adult patients with ADHD who were undergoing ADHD medication treatment at the outpatient departments of two university hospitals, one psychiatric hospital, and five outpatient clinics. We also recruited medical professionals who routinely treated adult patients with ADHD to review the DA prototype and complete the questionnaire.

For each group, approximately 20 individuals were approached. The sample size was selected in accordance with the methods used in the DA literature for previous acceptability testing.^26^, ^27^ We asked both patients and healthcare professionals to review the DA prototype and complete the survey.

The results were used to modify and improve the DA prototype to develop a final version that would be acceptable for use in a clinical setting. The effectiveness of the final DA among individuals in a dilemma regarding continuation or discontinuation of ADHD medications was not tested in the field as that was not the aim of this study.

### Data analysis method

Data are presented using descriptive analysis such as mean, standard deviation, percentage, and proportion. Qualitative data are presented verbatim from the participants.

## RESULTS

### Components of the DA prototype

**FIGURE 2:**
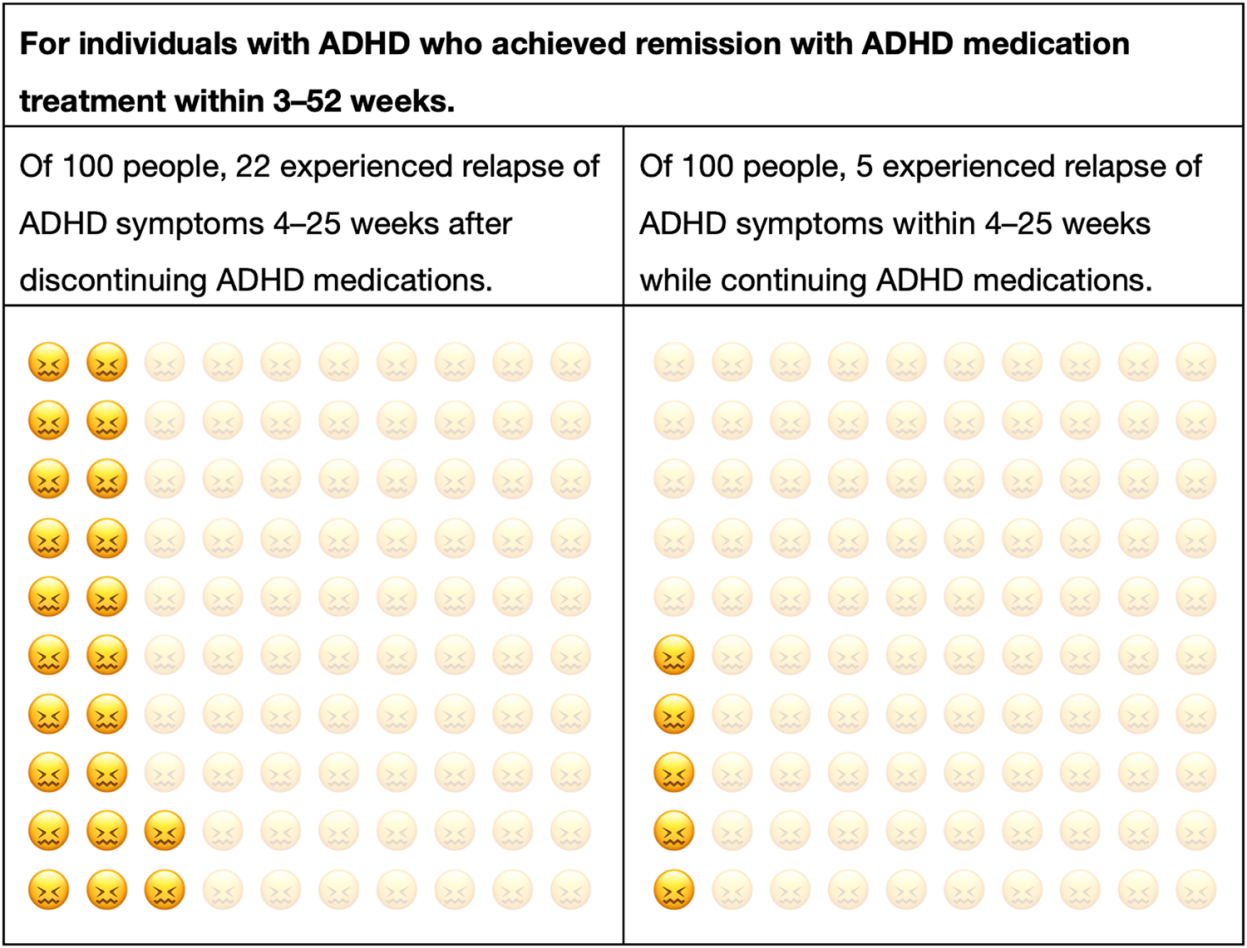
Pictorial diagram of outcomes of the DA for continuing or discontinuing ADHD medication. ADHD, attention‐deficit/hyperactivity disorder; DA, decision aid.

The developed DA prototype comprised a 28‐page A5 paper booklet. The introductory part of the book consisted of a description of the target population, information on how to use the DA, description of ADHD, and information on pharmacological and nonpharmacological treatments for ADHD, such as environmental adjustment, behavioral therapy, and psycho‐education. The DA prototype also contained options for continuing (option 1) and discontinuing (option 2) ADHD medications, the pros and cons of each option, and a value clarification exercise for each option. The outcomes of these options were based on our meta‐analysis, which showed that discontinuing ADHD medication was associated with the relapse of ADHD symptoms in adult patients, with a statistically significant RR of 3.70.^24^ To visualize these outcomes in the DA prototype, we used pictorial diagrams that consisted of 100 faces, with some faces shaded to represent the proportion of people predicted to experience the outcomes (Figure 2). In addition to presenting the pictorial diagrams, we also explained that the analysis of the relapse showed a statistically significant RR in adult patients with ADHD. The DA prototype also included a memo field in which individuals could suggest any additional comments or questions, which can be used during consultation on the discontinuation of ADHD medications. Additionally, the DA provided information for gradual tapering when discontinuing ADHD medications.

In the appendices of the DA prototype, we used the Questionnaire Adult ADHD with Difficulties (QAD)^28^ and Weiss Functional Impairment Rating Scale Self‐Report (WFIRS‐S)^29^ to provide information on various types of nonpharmacological treatments such as lifestyle and behavioral changes, frequently asked questions on ADHD treatment, and self‐rating scales of functional impairments. Finally, we added information targeting children and adolescents with ADHD.

The contents of the DA prototype are summarized in Supporting Information 1: Table S1.

### Acceptability testing

#### Patients

All 20 adult patients with ADHD who were undergoing ADHD medication treatments reviewed the DA prototype and completed the mixed‐method questionnaire. The mean age of the participants was 33.3 ± 11.8 years, and they comprised seven women and 11 men (two unknown). Five patients (25%) had a high‐school degree or lower level of education, two (10%) had vocational college‐level education, and 13 (65%) were university graduates.

The results of the four Likert scales that assessed the way information was presented in each section of the DA prototype were generally favorable (Table 1 **)**. Other responses of participants were as follows: 17 of 20 (85%) participants responded that the length of the presentation was just right, 15 of 20 patients (75%) rated the amount of information as just right, 11 of 19 patients (53%) responded that the presentation was well balanced, 14 of 20 patients (70%) responded that the DA was useful for deciding on the continuation or discontinuation of ADHD medication, 12 of 20 patients (60%) responded that the DA enabled foresight into the outcomes of the two options, 15 of 17 patients (88%) responded that the DA facilitated the decision, and 13 of 17 patients (76%) deemed that the DA included enough information to help make the decision to continue or discontinue ADHD medications.

**TABLE 1:** Service users' assessment of the way information is presented in each section of the decision aid prototype (*n* = 20)

	Mean	SD
About this booklet/instructions on use	2.65	0.87
What is ADHD?	2.70	0.73
Further treatment options	2.70	0.66
Comparison of the pros and cons of each option	2.85	0.67
Comparison of the consequences of each option	2.65	0.75
Value clarification	2.65	0.81
Preparation for shared decision making	2.55	0.69
(When discontinuing medication) Further treatment options	2.65	0.81
Appendices (*n* = 18)	2.72	0.83

*Note*: Rating system: four‐point Likert scale from 1 to 4: 4, excellent; 3, good; 2, fair; 1, poor.

Abbreviations: ADHD, attention‐deficit/hyperactivity disorder; SD, standard deviation.

The narrative feedback included positive comments on the DA prototype from patients. Some examples are provided below:

The pros and cons of each option were put in a matrix, which was easy to understand.

Presenting the pictorial diagrams with faces was very easy to understand.

It was a good opportunity to learn important information regarding my treatment.

I learned things about ADHD medications that was prescribed for me.

I like this DA because it includes a memo field in which I can make comments or questions which could be used during consultation.

The information in the appendices, such as self‐rating scales, is very useful.

I felt uneasy feeling because it might induce me to discontinue ADHD medications.

Furthermore, there were suggestions to provide additional explanations of some terms.

#### Healthcare providers

All 19 psychiatrists who were invited to take part in the study reviewed the booklet and completed the questionnaire. The mean age was 42.4 ± 8.3 years, and they comprised seven women and 12 men.

The perception of the prototype among the healthcare professionals was also favorable overall (Table 2). The psychiatrists attested to several strengths of the DA prototype: the overall concept, visualization, and friendliness of the information, simple language, and well‐balanced information for each option. Moreover, the feedback included recommendations for improving the DA, which are provided below:

Because of too many words, more figures/tables should be used.

There are too many words on the DA prototype, it would be better to reduce the total words.

In the instructions section, it would be better to recommend the need for patients to discuss options with family members during deliberation.

**TABLE 2:** Perceptions of the decision aid prototype among service providers (*n* = 19)

	Mean	SD
It will be easy for me to use.	3.37	1.07
It is easy for me to understand.	3.37	1.01
It will be easy for me to experiment by using the strategy before making a final decision to adopt it.	3.32	0.89
The results of using the strategy will be easy to see.	3.47	0.96
This strategy is better than how I usually go about helping patients decide about tapering ADHD medications (*n* = 18)	3.50	1.20
This strategy is compatible with the way I think things should be done.	3.42	1.12
The use of this strategy is more cost‐effective than my usual approach to helping patients decide about tapering ADHD medications (*n* = 18).	3.11	1.18
Compared with my usual approach, this strategy will result in my patients making more informed decisions.	4.00	1.05
Using this strategy will save me time.	2.89	1.15
This strategy is a reliable method for helping patients make decisions about tapering ADHD medications.	3.89	0.99
Pieces or components of the strategy can be used by patients.	3.37	1.01
This type of strategy is suitable for helping patients to make value‐laden choices.	3.21	1.08
This strategy complements my usual approach.	2.84	1.30
Using this strategy does not involve making major changes to the way I usually do things.	2.84	1.30
There is a high probability that using this strategy may cause/result in more benefit than harm.	3.95	0.91

*Note*: Possible scored range from 1 = strongly disagree to 5 = strongly agree.

Abbreviations: ADHD, attention‐deficit/hyperactivity disorder; SD, standard deviation.

### Modifying the prototype by incorporating feedback

The DA steering committee reviewed the results of the acceptability testing. We discussed the trends in the responses and narrative feedback, and took the opportunity to improve the DA prototype.

### Developing the final DA

We revised the final DA, which contributed to a high‐quality DA (Supporting Information 2). The developed DA met all the IPDAS qualifying criteria (six of six) (Supporting Information 3: Table S2), criteria required for DA consideration (18 of 19), and all certification criteria (six of six) that deem the DA to have a low risk of harmful bias.^21^ Furthermore, the DA met most of the IPDAS quality criteria (19 of 23), which strengthens the DA, but whose omission does not present a high risk of harmful bias. The IPDAS criteria met by our DA were highly rated compared with other available Ottawa DAs that address healthcare decisions.^30^

Furthermore, the healthcare providers who will be using this tool will need to be informed regarding its correct usage. We therefore developed a manual for healthcare providers to describe DA content and how to use this tool in clinical settings (Supporting Information 4).

## DISCUSSION

In this study, we developed a DA for adult patients with ADHD which contained two options: whether to continue or discontinue ADHD medications after remission. The developed DA was validated by a steering committee that comprised experts on ADHD and DA methodology, including psychiatrists, a psychiatric pharmacist, and a psychiatric nurse. We assessed the acceptability of the DA among patients and healthcare providers. The responses of patients with ADHD were positive in almost all the questions, and healthcare providers also supported the use of our DA in clinical settings. Thus, the DA developed in this study can facilitate SDM between adult patients with ADHD and their healthcare providers when considering continuation or discontinuation of ADHD medications.

Our results are partly consistent with that of a previous study by Ibrahim & Donyai^15^ that developed DAs for parents, adolescents, and children regarding the decision on whether to embark on a planned drug holiday from methylphenidate. They reported the acceptability of their DAs with two panels, which were composed of child and adolescent mental health practitioners and the parents of children and adolescents with ADHD. They envisaged that the developed DAs would open up opportunity for the discussion of drug holidays for ADHD during annual reviews in line with the NICE guidelines.^11^

In our study, we developed a DA for adult patients with ADHD that focused on any preferences to stop medication treatment after remission. This focus forms the strength of our study by providing optional decisions on whether to stop ADHD medications for adult patients with ADHD. Ibrahim and Donyai^15^ discussed the possibilities that their DAs for children and adolescents with ADHD might involve decisions about stopping medication in events in which the overall balance of benefits and harms of discontinuation needs to be assessed. A previous study on DA for adult patients with ADHD involved decisions about further treatments but did not contain options for stopping medication after remission.^13^ To the best of our knowledge, no study has developed a DA for adult patients regarding the continuation or discontinuation of ADHD medications.

Another strength of our study was the inclusion of several ADHD medications, such as methylphenidate, lisdexamfetamine, atomoxetine, and guanfacine, in our DA. Although methylphenidate is the most commonly used ADHD medication in most countries, the prevalence of other ADHD medications has increased in recent times among adult patients with ADHD worldwide.^18^ Furthermore, the long‐term safety and effectiveness of ADHD medications in adult patients remain equivocal.^5^, ^31^ A recent NICE guideline for ADHD recommends that healthcare professionals should consider stopping ADHD medications when the assessment of the overall balance of benefits and harms suggests it to be appropriate.^11^ Our DA provides options for adult patients with ADHD to discuss any preferences to stop or continue ADHD medication. Moreover, we systematically developed a DA according to the IPDAS criteria. The IPDAS recommended the inclusion of tools, such as worksheets or question lists, for discussing options with practitioners. Our DA contained memo fields to prepare for decision‐making consultations. The DA also provided step‐by‐step instructions for adult ADHD patients to assist in decisions made with healthcare providers to continue or discontinue medication after ADHD remission. This is one possible application for creating the DAs.

There were several limitations in this study. First, we included adult patients with ADHD who experienced remission with ADHD medications. However, it might be difficult to determine ADHD remission because improvement in symptoms correlates moderately, but not significantly, with improvement in daily function and overall quality of life.^2^ Thus, to evaluate functional improvement in adult patients with ADHD, we provided self‐rating scales of functional impairments (QAD and WFIRS‐S) in the appendices of the DA. Second, the sample size was relatively small, which might have implications for the generalizability of the results. Third, although our DA met most of the IPDAS quality criteria,^21^ several other criteria may be important in improving the DA, including field testing and evidence‐based outcomes. Fourth, we excluded adults with ADHD who had other neurodevelopmental or psychiatric disorders as comorbidities. Several adults with ADHD have at least one comorbidity in clinical practice.^31^ However, limited evidence is available concerning differences in pharmacological treatment efficacy and safety for adults with ADHD with and without comorbidities. A recent systematic review showed that the efficacy and safety of pharmacological treatments may differ in children and adolescents with ADHD depending on the comorbidities.^32^ To clarify the utility of a DA for deciding on the continuation or discontinuation of ADHD medications after remission in adult patients, we excluded adults with ADHD who had comorbidities. Further studies are required to develop a DA for adults with ADHD and comorbidities. Fifth, the sample size was relatively small. We determined the sample size based on previous investigations into the acceptability of DAs.^13^, ^22^ Another limitation was the lack of several demographic or clinical characteristics, such as IQ or socioeconomic status. The relatively small sample size and the lack of clinical information on patients may affect the results concerning the acceptability of the developed DA. Finally, the effectiveness of the final DA among adult patients with ADHD was not tested. Testing with a larger sample is needed to confirm the effectiveness of the DA in clinical settings. Few studies have examined the effectiveness of DAs among adult patients with ADHD. Further studies should field test the DA during the SDM process with individuals with ADHD and their healthcare providers to verify the efficacy of the DA.

## CONCLUSION

We successfully developed a DA for adult patients with ADHD who might be faced with decision‐making challenges on whether to continue or discontinue ADHD medication after remission. Our results could facilitate the SDM process between patients and their healthcare providers regarding continuing or discontinuing ADHD medications following remission. In addition, we included information targeting children and adolescents with ADHD in the DA. With further confirmation of the findings, our study may lead to the development of individualized medical treatment for individuals with ADHD.

## AUTHOR CONTRIBUTIONS

All authors share responsibility for the final version of the work submitted and published.

## CONFLICT OF INTEREST

The authors declare no conflicts of interest.

## ETHICS APPROVAL STATEMENT

All study participants provided informed consent. This study was approved by the ethics review board of Kyorin University, Tokyo, Japan (approval number R02‐037).

## PATIENT CONSENT STATEMENT

All study participants provided informed consent.

## CLINICAL TRIAL REGISTRATION

N/A.

## Supporting information

Supporting information.

## Data Availability

All relevant data are within the paper.
