# Supplementary material for: Decision aid development and its acceptability among adults with attention‐deficit/hyperactivity disorders regarding treatment discontinuation after remission
Source: PCN Rep. 2022 Nov 14;1(4):e57. doi: 10.1002/pcn5.57 (PMC11114424; doi:10.1002/pcn5.57)
Supplement: Supplementary file 1 — Supporting information. [file PCN5-1-e57-s001.pdf]

Supporting Information 1

Contents of the decision aid for adult patients with attention-deficit/hyperactivity disorder (ADHD) regarding continuation or discontinuation of ADHD medications

Contents	Pages
**About this booklet**	
● Description of decisions to be considered<br>● Description of the target population<br>● Instructions on the use of this booklet	1–2
**What is attention-deficit/hyperactivity disorder (ADHD)?**	
● Objective information on ADHD, such as clinical symptoms and treatments of ADHD	3
**Step 1: Further treatment options**	
● Continuation of ADHD medications and information on ADHD medications (advantages and disadvantages of each drug category)<br>● Discontinuation of ADHD medications and information on nonpharmacological interventions, such as environmental adjustments, behavioral interventions, or psychological interventions	4–5
**Value clarification**	
● Options provided: continuing or discontinuing ADHD medications	6
**Step 2: Comparison of the features of each option**	
● A table comparing the features of each option (advantages, disadvantages, and consequences)<br>● Pictorial diagrams comparing the consequences of each option	7–8
**Step 3: Value clarification**	
● A value clarification exercise using a five-point Likert scale	9
**Step 4: Preparation for shared decision making**	
● Memo field preparation for decision-making consultation	10
**(If discontinuing medications) Step 1: Further treatment options**	
● Information on nonpharmacological interventions, such as environmental adjustments, behavioral interventions, or psychological interventions	11
**(If discontinuing medications) Step 2: How to stop a medication**	
● Information on how to stop each medication (methylphenidate, lisdexamfetamine, atomoxetine, and guanfacine)	12
**Appendix 1: Lifestyle and behavioral changes for controlling ADHD symptoms**	
● Information on behavioral interventions	14–16
**Appendix 2: Self-rating scales of functional impairments for adult patients with ADHD**	

• Questionnaire Adult ADHD with Difficulties<br>• Weiss Functional Impairment Rating Scale Self-Report	17–20
**Appendix 3: Frequently asked questions and answers**	
• Frequently asked questions and answers regarding sleep medication	21–22
**Appendix 4: How to use this booklet for children and adolescents with ADHD**	
• Instructions on the use of this booklet for children and adolescents with ADHD	23–24

CONTINUING **TAPERING** **STOPPING**

A **decision aid** for ADHD

Regarding discontinuation of medications

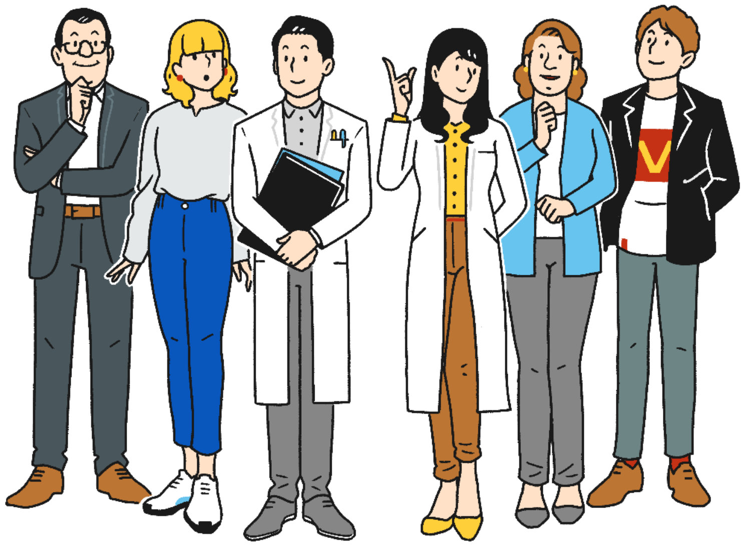

This decision aid is based on the Guidelines for the Appropriate Use and Withdrawal of ADHD Medication and the Guidelines for Psychotropic Drug Discontinuation Strategies

Table of Contents

About this booklet 1

How to use it? 2

What is ADHD? 3

Further future treatment options for

  Continuing taking ADHD medications, and 4

  Non-pharmacological treatments 5

  Value clarifications 6

    Option 1 『Continuing ADHD medications』

    Option 2 『Discontinuing ADHD medications』

Comparison of the features of each option 7

Comparison of the consequences of each option 8

Value clarifications 9

Preparations for shared decision making 10

 (If discontinuing medications)

  Further treatment options 11

  How to stop a medication 12

Appendix 1: Lifestyle and behavioral changes for controlling ADHD 14

Appendix 2: Self-rating scales of functional impairments 17

Appendix 3: Frequently Asked Questions and Answers 21

Appendix 4: How to use this for children and adolescents with ADHD 23

About this decision aid

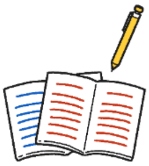

This decision aid is designed to assist individuals with attention-deficit/hyperactivity disorder (ADHD) who are using ADHD medications, to determine further treatment with healthcare providers.

There are two options: continuing and discontinuing ADHD medications. However, each treatment method has its pros and cons. For this reason, shared decision making, which reflects the individuals' preferences and values in treatment, is recommended.

Therefore, we have prepared this aid to help individuals and their healthcare providers choose the appropriate method by comparing the pros and cons of continuation or discontinuation of ADHD medications.

【Those who are eligible to use this guide】

・Adults who have been taking one ADHD medication and have experienced remission with ADHD medication treatment.

【Those who are not eligible to use this guide】

・Individuals who (1) failed to respond to ADHD medications and did not experience remission with those medications, (2) achieved remission in less than 3 months, and (3) have any other neurodevelopmental or psychiatric disorders.

In the event of uncertainty regarding the eligibility for this aid, the healthcare provider should be consulted.

How to use this decision aid

The decision aid is meant for discussion with healthcare providers on the determination of the future treatment. You can take this aid home, read it carefully, and discuss it with your family members; thus, the future treatment plan may be deliberated together.

This decision aid should be read carefully

Answers should be marked using a circle or by filling the memo fields.

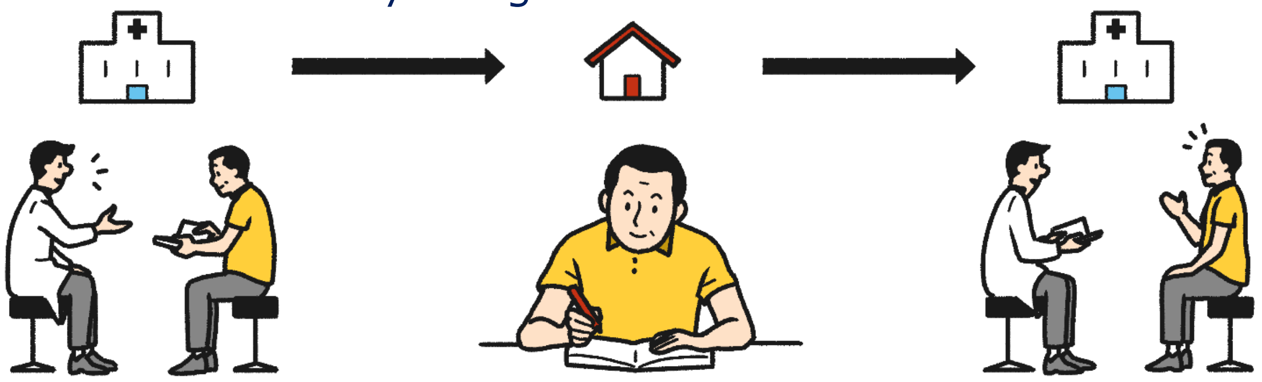

During consultation

- Your current condition will be explained
- Treatment options will be reviewed

During consultation

- The circled items and the contents of the memo should be discussed.
- Further treatment should be planned.

※If a decision cannot be made at once, the decision aid should be brought back for further consideration.

What is ADHD?

Attention-deficit/hyperactivity disorder (ADHD) is a neurodevelopmental disorder defined by impairing levels of inattention, disorganization, and/or hyperactivity-impulsivity. These symptoms are present prior to 12 years of age and in two or more settings (e.g., at home, school, work).

The treatment for individuals with ADHD can be pharmacologic, nonpharmacologic, or both. Pharmacological treatment is usually reserved for severe cases or for milder cases that do not respond to nonpharmacological treatments.

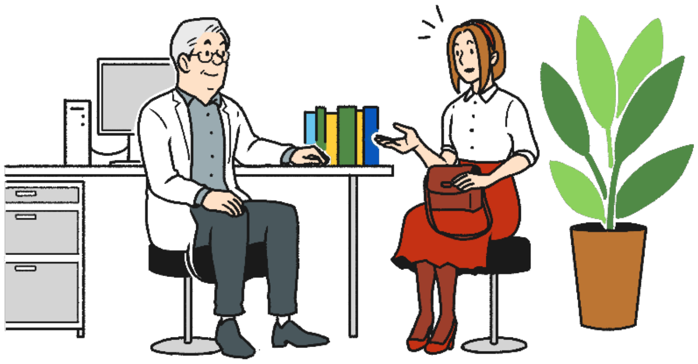

In the following section, we will look at further treatment options when ADHD has improved. ☞

Step 1 Each option should be understood.

What are ADHD medications?

ADHD medications increase the activity of neurotransmitters such as dopamine and noradrenaline and increase brain activity in people with ADHD.

Psychostimulants

Drugs:

OROS methylphenidate【Concerta Tablets®】

Lisdexamfetamine【Vyvanse Capsules®】*

*In Japan, not approved for adults; can be continued only if initiated before 18 years of age.

Characteristics

The effect can last for half a day.

Loss of appetite, weight loss, insomnia, increased blood pressure/heart rate may be observed.

There is a risk of dependence and abuse, and distribution is regulated.**

** You must provide proof of identity when you receive prescriptions.

Non-stimulants

Drugs:

Atomoxetine【Strattera Capsules®】

Guanfacine 【Intuniv Tablets®】

Characteristics

The effect can last all day. Low risk of dependence/abuse.

Loss of appetite, nausea, increased blood pressure, tachycardia, etc. may be observed.

[Atomoxetine] There is a generic preparation with a choice of liquid preparations.

[Guanfacine] Decreased blood pressure, sedation/drowsiness, headache, etc.

Frequently asked questions and answers ➡ Appendix 3

What are nonpharmacological treatments?

・Environmental adjustments

Environmental adjustments can help make it easier to live and use your abilities. Prepare the environment and gain an understanding of the surroundings.

・Behavioral intervention

Learn about the characteristics of ADHD and devise ways to live with it.

・Psychological intervention

Devise ways to get involved to increase the desired behaviors of people with ADHD want to learn.

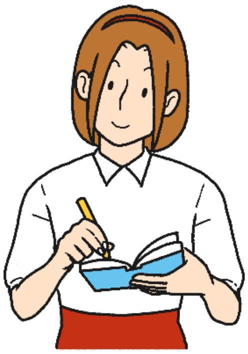

Further treatment options

What you can do to lead a better daily life other than drug therapy which will continue regardless of future treatment options (Appendix 1)?

Option 1: Continuing ADHD medications

Option 2: Discontinuing ADHD medications

Regardless of the choice you make, regular review of your treatment is necessary (Appendix 2).

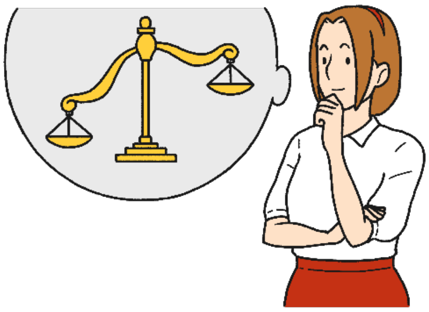

Next, let us compare the advantages and disadvantages of each option ☞

Pros and Cons of Each Option

Step 2 Compare the pros and cons of each option

	Option 1<br>**Continuing ADHD medications**	Option 2<br>**Discontinuing ADHD medications**
**Pros** ☺	• Does not exacerbate ADHD symptoms<br>• Does not deteriorate daily function or quality of life	• Eliminates side effects<br>• Reduces financial burden<br>• Reduces the burden of going to the hospital
**Cons** ☹	• Side effects persist<br>• Current financial burden continues<br>• Current burden of going to the hospital continues	• May worsen ADHD symptoms<br>• May deteriorate daily function and quality of life

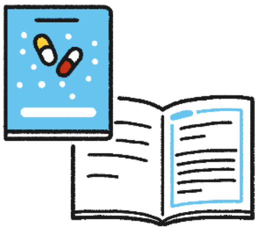

Consequences of each option

Here are our estimates of the consequences of continuing/discontinuing medications in adults with ADHD. Each face represents one person and shows how many people out of 100 would experience ADHD relapse.

**Option 1**<br>**Continuing ADHD medications**	**Option 2**<br>**Discontinuing ADHD medications**

For adults who are diagnosed with ADHD, treated with ADHD medications, and remitted for 3–52 weeks.

Of 100 people, 5 experienced relapse of ADHD symptoms while continuing ADHD medications within 4–25 weeks.

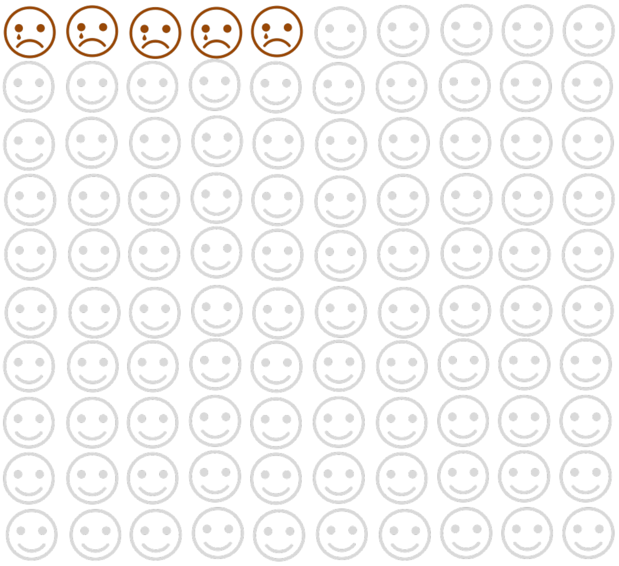

Of 100 people, 22 experienced relapse of ADHD symptoms after discontinuing ADHD medications within 4–25 weeks.

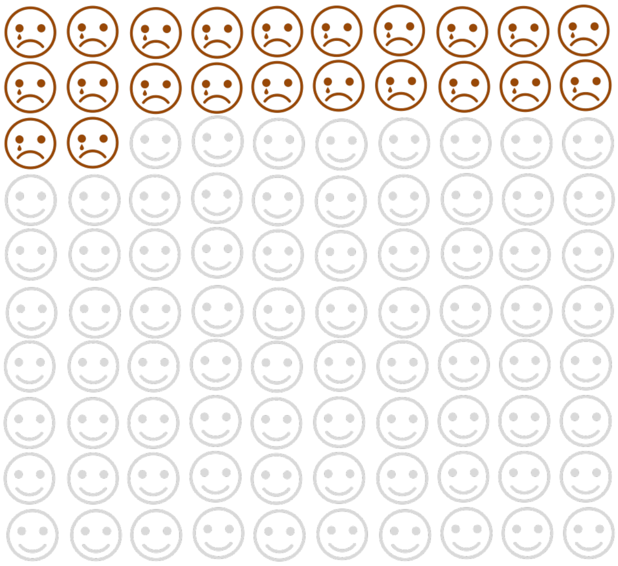

There was a statistically significant difference in the rate of exacerbation of ADHD symptoms.

What is important to you?

Step 3 Organize what is important

Below, we have listed the main reasons for choosing each option. The importance of each option to you should be analyzed. The numbers that are applicable to your importance should be circled.

Option 1 Reason for continuing ADHD medications

	Not important Important					
e.g., Avoid exacerbation of symptoms	0	1	2	3	4	5
Other (please state freely)						
·	0	1	2	3	4	5
·	0	1	2	3	4	5
·	0	1	2	3	4	5
·	0	1	2	3	4	5

Option 2 Reason for discontinuing ADHD medications

	Not important Important					
e.g., Avoid side effects	0	1	2	3	4	5
Other (please state freely)						
·	0	1	2	3	4	5
·	0	1	2	3	4	5
·	0	1	2	3	4	5
·	0	1	2	3	4	5

Preparing for a consultations

Step 4 Preparing for the discussion with the doctor

Depending on the thoughts and weightage, we can discuss
option 1: continuing ADHD medications and
option 2: discontinuing ADHD medications.

Please note down any current feelings and thoughts, including any questions or concerns.

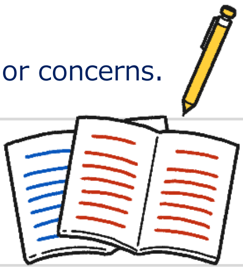

(If discontinuing medications)

Further treatment options

Step 1 Continue with the more conscious ingenuity of daily life that has been effective so far.

Environmental adjustments

・ If you have too many tasks to do, or if you have difficulty prioritizing them, organize them one by one, or organize them in a memo before working on them.

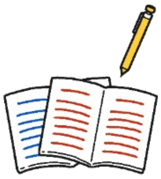

Psychological intervention

・Stopping a medication does not mean that you no longer have ADHD. Try to live a life that is more commensurate with your strengths and weaknesses than before.

Behavioral intervention

・ Withdrawal of medication may cause ADHD symptoms to reappear even when you are already doing your best. Family members should acknowledge and encourage individuals with ADHD and desist from using negative language.

(If discontinuing medications)

How to stop a medication

Step 2 Choose the discontinuation method that suits your prescription drug

Concerta Tablets® or Vyvanse Capsules®

Carefully watch for the worsening of ADHD symptoms while reducing the dose or gradually increasing drug holidays.

Strattera Capsules®

It is possible to discontinue this medication without gradually reducing its dose. However, to avoid anxiety and effects on blood pressure, etc., gradually reduce the dose and check for deterioration of ADHD symptoms.

Intuniv Tablets®

Sudden discontinuation may cause increased blood pressure and tachycardia. Therefore, slowly reduce the dose. Please consult with your doctor about the method for discontinuation.

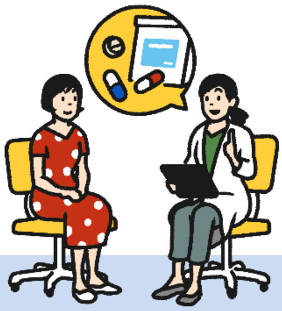

Appendix 1

Lifestyle and behavioral changes for controlling ADHD symptoms

Appendix 2

Self-rating scales of functional impairments in adult patients with ADHD

Appendix 3

Frequently asked questions and answers

Appendix 4

Instructions on the use of this booklet for children and adolescents with ADHD

Appendix 1: **Lifestyle and behavioral changes for the control of ADHD symptoms**

You do not have to improve everything at once.
Choose one or two tasks from what you can do and work on them for a certain period of time!

● For individuals who have trouble with cleaning up in daily life

If you aim for perfection, you will face difficulties in reaching your goal.

First, do not aim for perfection; instead, start with what you can do.

For example, reduce the number of tasks you have at a time so that the place to be tidied up matches the targeted place ; mark the places of things so it is easy to put them back later; or set the timer to 15 minutes a day and work on the required tasks in that time. There are also ways to divide the time and find a suitable way to concentrate in a short time.

When tasks go wrong, take the opportunity to encourage yourself or take the plunge and let go of what you could not do. As a measure against frustration, it would be good to perform a calming activity (securing a cool-down) according to your taste, such as exercising or listening to music.

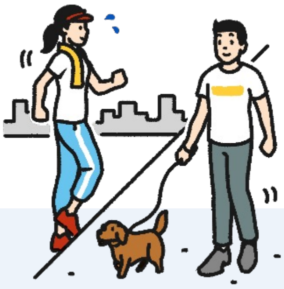

● **For individuals who have trouble managing tasks at school or work.** If you have multiple tasks and you try to do all of them at once, they will be halfway accomplished.

First, let us divide the work into small parts and do them one by one. Write down your priorities and do what you need to do in the order of priority.

If things go wrong, it may be effective to divide the work into even smaller parts and then write down the priorities. It may be effective also to set priorities with a TO DO management app, break down large tasks into small steps, and lower the hurdles to work on.

● **For individuals who are forgetful in their daily lives, school and work**

If you cannot stop forgetting items even if you recheck your belongings and plan in advance, increase the number of times that you check them.

First of all, try to put the items that you need in a place that you pass by every day, such as near the entrance or door. Basically, you decide where to put it, but if it is a key, you can attach it to an iron door with a magnet sheet or use a key chain to link it with a bag.

When things go wrong, take time to organize the situation at the beginning and end of work, reduce the amount of work taken out of the workplace (by leaving writing instruments and notebooks at the workplace). As a countermeasure to forgetting, it may be effective to give a spare document to someone who is good at organizing.

● For individuals who cannot keep their promises and deadlines at work or in relationships

To meet the deadline, it is effective to make the schedule concrete.

First, make the schedule an easy-to-understand table and put it in a conspicuous place. Let us use the reminder function linked with the calendar app, or use a schedule management app or software (use of high-tech equipment).

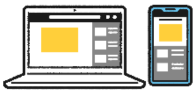

If things go wrong, it may be useful to make a plan with in-built breaks or to consult with the people around you before making promises.

● For individuals who tend to talk excessively, who easily get frustrated, or who feel negative feelings

If you have something to worry about or do not like, you may talk excessively or explode. You may also feel guilty or embarrassed.

If you feel that this is a hindrance to your relationships or work, it is a good idea to take a breath before expressing your feelings. It is also necessary for an individual to take a break and leave the place at once. In addition to checking your feelings on a daily basis, it is important to find tips to deal with anger well (anger management) and to change your mood to eliminate frustration.

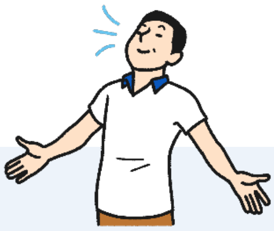

Appendix 2 **Self-rating scales of functional impairments in adult patients with ADHD**

If symptoms worsen, strengthening psychosocial treatment or resuming medication may be an option.

Questionnaire Adult ADHD with Difficulties

Questionnaire Adult ADHD with Difficulties (QAD) is a tool for assessing daily living functions in adult patients with ADHD so that they can understand the situations in their daily lives that can pose difficulties because of ADHD symptoms.

In situations where adult patients with ADHD are not good at a task or find it difficult, the QAD allows the evaluation of living functions along with the flow of the day, from waking up to going to bed.

By continuing to check for this regularly from the beginning of ADHD treatment, you will see how much your life's difficulties have improved.

Weiss Functional Impairment Rating Scale Self-Report

Weiss Functional Impairment Rating Scale Self-Report (WFIRS-S) is a tool for assessing where an adult ADHD patient is facing difficulties in daily life. The target life scenes are the seven areas of family, workplace, school, life skills, self-concept, socializing, and risk. By continuing to check WFIRS-S regularly from the beginning of ADHD treatment, you will also see how much your life's difficulties have improved.

Questionnaire Adult ADHD with Difficulties[2,3]

Please fill in the blanks with the date, the date of the next medical examination, etc., and check the symptoms you are currently feeling in four steps while remembering the flow of the day. After taking this assessment, please show it to your doctor at the next consultation.

Today's date	Next consultation day	Administration	Dosage
/ /	/ /	/day	mg/day

0 = completely disagree 1 = slightly agree 2 = quite agree 3 = exactly agree

		0	1	2	3
Morning	1. Can you get up from bed quickly in the morning?	0	1	2	3
	2. After waking up in the morning, can you quickly get dressed (washing your face, brushing your teeth, changing clothes, etc.)?	0	1	2	3
	3. Can you finish breakfast promptly?	0	1	2	3
	4. Have you been able to spend the morning without any troubles or quarrels with those around you?	0	1	2	3
Daytime	5. Can you start work or housework smoothly?	0	1	2	3
	6. Are you able to concentrate on your work or housework in a well-planned manner, just like your surroundings?	0	1	2	3
	7. Are your interpersonal relationships going well?	0	1	2	3
	8. Can you remember your promises, errands and jobs?	0	1	2	3
	9. Are you able to spend your time without losing something important?	0	1	2	3
	10. Can you wait slowly when you need it?	0	1	2	3
	11. Have you finished what you need to do?	0	1	2	3
	12. Are you able to spend time without talking excessively or engaging in some activity?	0	1	2	3
	13. Do you spend your time without being told that you are restless or noisy?	0	1	2	3
	14. Are you able to participate in your leisure activities without any problems?	0	1	2	3
Night	15. Do you spend your time without being overly absorbed in certain things (computers, mobile phones, games, slingshots, drinking, smoking, etc.)?	0	1	2	3
	16. Are you well prepared and can you wait?	0	1	2	3
	17. Is your sleep cycle stable?	0	1	2	3
Throughout the day	18. Do you feel confident without feeling depressed or anxious?	0	1	2	3
	19. Are you able to spend your time without any confusion or trouble?	0	1	2	3
	**Please enter the number of check items x points →**	0			
	**Please calculate the total score →**	Total score			

Weiss Functional Impairment Rating Scale Self-Report[4]

Please fill in the blanks with the date, the date of the next medical examination, etc., and check the symptoms you are currently feeling in four steps while remembering the flow of the day. After taking this assessment, please show it to your doctor at the next consultation.			
Today’s date	Next consultation day	Administration	Dosage
/ /	/ /	/day	mg/day

0 = Never or not at all 1 = Sometimes or somewhat
2 = Often or much 3 = Very often or very much ☐ = n/a

		0	1	2	3	☐
A FAMILY	1. Having problems with family	0	1	2	3	☐
	2. Having problems with spouse/partner	0	1	2	3	☐
	3. Relying on others to do things for you	0	1	2	3	☐
	4. Causing fights in the family	0	1	2	3	☐
	5. Making it hard for the family to have fun together	0	1	2	3	☐
	6. Problems with taking care of your family	0	1	2	3	☐
	7. Problems with balancing your needs against those of your family	0	1	2	3	☐
	8. Problems with losing control with family	0	1	2	3	☐
B WORK	1. Problems with performing required duties	0	1	2	3	☐
	2. Problems with getting your work done efficiently	0	1	2	3	☐
	3. Problems with your supervisor	0	1	2	3	☐
	4. Problems with keeping a job	0	1	2	3	☐
	5. Getting fired from work	0	1	2	3	☐
	6. Problems with working in a team	0	1	2	3	☐
	7. Problems with your attendance	0	1	2	3	☐
	8. Problems with being late	0	1	2	3	☐
	9. Problems with taking on new tasks	0	1	2	3	☐
	10. Problems with working to your potential	0	1	2	3	☐
	11. Poor performance evaluations	0	1	2	3	☐
C SCHOOL	1. Problems with taking notes	0	1	2	3	☐
	2. Problems with completing assignments	0	1	2	3	☐
	3. Problems with getting your work done efficiently	0	1	2	3	☐
	4. Problems with teachers	0	1	2	3	☐
	5. Problems with school administrators	0	1	2	3	☐
	6. Problems with meeting minimum requirements to stay in school	0	1	2	3	☐
	7. Problems with attendance	0	1	2	3	☐
	8. Problems with being late	0	1	2	3	☐
	9. Problems with taking on new tasks	0	1	2	3	☐
	10. Problems with working to your potential	0	1	2	3	☐
	11. Problems with inconsistent grades	0	1	2	3	☐

0 = Never or not at all 1 = Sometimes or somewhat
2 = Often or much 3 = Very often or very much ☐ = n/a

		0	1	2	3	☐
D LIFE SKILLS	1. Excessive or inappropriate use of internet, video games or TV	0	1	2	3	☐
	2. Problems with keeping an acceptable appearance	0	1	2	3	☐
	3. Problems with getting ready to leave the house	0	1	2	3	☐
	4. Problems with getting to bed	0	1	2	3	☐
	5. Problems with nutrition	0	1	2	3	☐
	6. Problems with sex	0	1	2	3	☐
	7. Problems with sleeping	0	1	2	3	☐
	8. Getting hurt or injured	0	1	2	3	☐
	9. Avoiding exercise	0	1	2	3	☐
	10. Problems with keeping regular appointments with doctor/dentist	0	1	2	3	☐
	11. Problems with keeping up with household chores	0	1	2	3	☐
	12. Problems with managing money	0	1	2	3	☐
E SELF-CONCEPT	1. Feeling bad about yourself	0	1	2	3	☐
	2. Feeling frustrated with yourself	0	1	2	3	☐
	3. Feeling discouraged	0	1	2	3	☐
	4. Not feeling happy with your life	0	1	2	3	☐
	5. Feeling incompetent	0	1	2	3	☐
F SOCIAL	1. Getting into arguments	0	1	2	3	☐
	2. Trouble cooperating	0	1	2	3	☐
	3. Trouble getting along with people	0	1	2	3	☐
	4. Problems having fun with other people	0	1	2	3	☐
	5. Problems with participating in hobbies	0	1	2	3	☐
	6. Problems with making friends	0	1	2	3	☐
	7. Problems with keeping friends	0	1	2	3	☐
	8. Saying inappropriate things	0	1	2	3	☐
	9. Complaints from neighbors	0	1	2	3	☐
G RISK	1. Aggressive driving	0	1	2	3	☐
	2. Doing other things while driving	0	1	2	3	☐
	3. Road rage	0	1	2	3	☐
	4. Breaking or damaging things	0	1	2	3	☐
	5. Doing things that are illegal	0	1	2	3	☐
	6. Being involved with the police	0	1	2	3	☐
	7. Smoking cigarettes	0	1	2	3	☐
	8. Smoking marijuana	0	1	2	3	☐
	9. Drinking alcohol	0	1	2	3	☐
	10. Taking "street" drugs	0	1	2	3	☐
	11. Sex without protection (birth control, condom)	0	1	2	3	☐
	12. Sexually inappropriate behavior	0	1	2	3	☐
	13. Being physically aggressive	0	1	2	3	☐
	14. Being verbally aggressive	0	1	2	3	☐

Appendix 3 **Frequently asked questions and answers**

● **Do I have to keep taking medications for the rest of my life?**

At this time, there are no clear criteria or recommendations as to how long you should continue to take ADHD treatment. Some experts believe that long-term drug continuation is desirable for improving ADHD symptoms. However, for those who continue to have their symptoms sufficiently alleviated, discontinuing ADHD treatment may be an option. Talk to your doctor.

● **Doesn't the effect of ADHD medications gradually weaken?**

If you take the medicine as instructed by your doctor, it is thought that the effect will not gradually weaken and the amount will not increase. Changes in the environment, such as employment and marriage, which increase social burden, may worsen ADHD symptoms. Even in such cases, the effectiveness of the drug does not diminish. Talk to your doctor about appropriate psychosocial treatment and environmental adjustments.

● **Can this booklet be used by children?**

See Appendix 4.

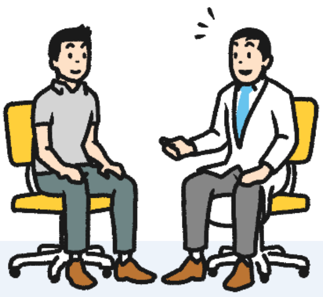

- **I want a child, can I continue to take medicine?**

The package insert states that Intuniv Tablets® should not be given to pregnant or potentially pregnant women. Concerta Tablets® should not be given to pregnant or potentially pregnant women. Strattera Capsules® and Vyvanse Capsules® can be given only if the therapeutic benefit outweighs the risk. If you find out that you are pregnant, talk to your doctor or pharmacist.

- **Is it okay to breastfeed while taking medication?**

It is known that ADHD drugs can be physiologically transferred to breast milk. The inserted package indicates that breastfeeding should be avoided while taking ADHD medications. ADHD medications could be discontinued to continue breastfeeding, so discuss with your doctor or pharmacist.

- **Others (Make a note of yours question to the doctor)**

  -
  -
  -
  -

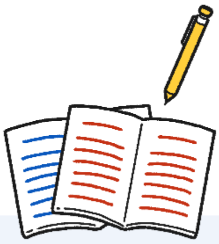

Appendix 4 **How to use this booklet for children and adolescents with ADHD**

For children, much of what is written in this booklet applies, but there are some differences. Because children are in the process of development, there are concerns about appetite loss, weight loss (no expected weight gain), and growth suppression (slow height growth) caused by drug therapy, especially psychostimulants. In reality, it is believed that the effect on height as an adult is extremely small. However, you may want to keep an eye on your child's weight and height growth; in particular, if your child experiences a loss of appetite, there will be a need to supplement it with snacks or take a drug holiday.

We show how often ADHD symptoms worsen when children who have experienced sufficient improvement in their symptoms over a period of time with ADHD medication stop taking the drug (see next page).

Regarding whether to continue or discontinue drug therapy, you can make a treatment decision by considering the advantages and disadvantages of each option and their importance to the child. However, the most important thing is who will make these decisions and how.

To make therapeutic decisions, you need to understand each option, anticipate the consequences of making a choice, and understand that you are free to make your own choices. However, it can be difficult for children. That is why parents should agree to ensure the best results for their children.

Option 1<br>**Continuing ADHD medications**	Option 2<br>**Discontinuing ADHD medications**

For **children and adolescents** who are diagnosed with ADHD, treated with ADHD medications, and remitted for 6 to 52 weeks.

Of 100 children and adolescents, 26 experienced relapse of ADHD symptoms while continuing ADHD medications within 6–36 weeks.

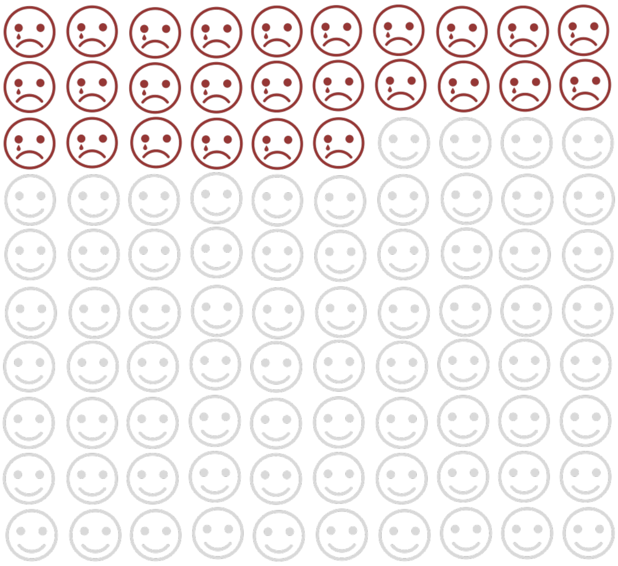

Of 100 children and adolescents, 48 experienced relapse of ADHD symptoms after discontinuing ADHD medications within 6–36 weeks.

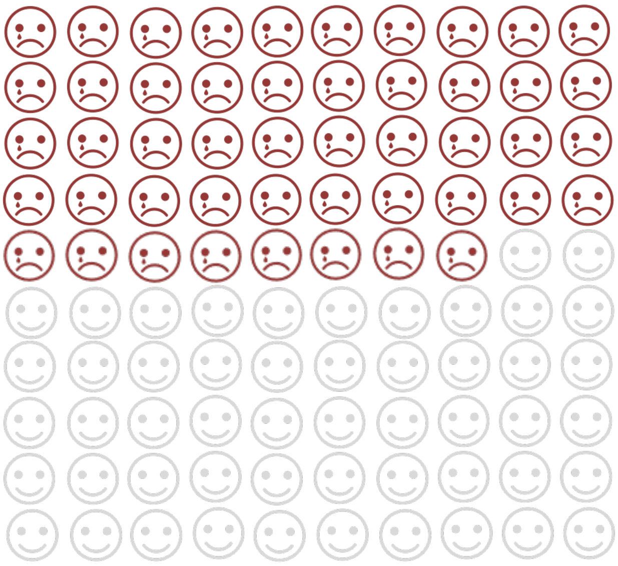

There was a statistically significant difference in the rate of exacerbation of ADHD symptoms.

In contrast, it is also important to explain the instruction in simple words as much as possible to enable children to understand within their abilities and to express their consent for the treatment options. The ability required for consent varies greatly depending on the child's age and intellectual ability, so this booklet cannot be used by children in the same way as adults. Take more time to talk with your doctor and make treatment choices that are convincing for both parents and children.

Conclusion

● Determine the right coping and treatment methods that are best for you

Each treatment option has its pros and cons. This decision aid is designed to help you understand these options well, discuss them with your healthcare providers while determining what is important to you, and make the choices that are right for you.

● Development of the decision aid

This decision aid was developed based on the responses and opinions of people who had been taking medications treatment for insomnia. The decision aid has also been checked by psychiatric specialists. This aid was developed with research grants from the Ministry of Health, Labor and Welfare of Japan (19GC1012).

● Updating the decision aid

This decision aid will be reviewed and updated as necessary.

※ The information provided here is intended to guide you in identifying the right coping or treatment method for you while you are consulting healthcare professionals; however, this decision aid is not intended to replace the advice provided by healthcare professionals.

References
1) Tsujii, N., Okada, T., Usami, M., Kuwabara, H., Fujita, J., Negoro, H., Kawamura, M., Iida, J., Saito, T. (2020). Effect of continuing and discontinuing medications on quality of life after symptomatic remission in attention-deficit/hyperactivity disorder. The Journal of Clinical Psychiatry, 81, 19r13015.
2) Questionnaire Adult ADHD with Difficulties (QAD) https://adhd.co.jp/pdf/Adult_QAD_checksheet.pdf
3) Inoue, K. (2019). A study on the reliability and validity of Questionnaire Adult ADHD with Difficulties (QAD). Bulletin of Living Science, 9-16.
4) Takeda, T., Tsuji, Y., Kanazawa, J., Sakai, T., Weiss, M.D. (2017). Psychometric properties of the Japanese version of the Weiss Functional Impairment Rating Scale: self-report. Attention Deficit and Hyperactivity Disorders, 9, 169-177.

Develop by Sleep Drug Group in Psychotropic Drugs Exit Strategy
Manual Research Group

Created: March 2021
Scheduled to updated: March 2023

This decision aid is based on the Guidelines for the Appropriate Use and Withdrawal of Sleep Medication and the Guidelines for Psychotropic Drug Discontinuation Strategies

©2021, Psychotropic Drugs Exit Strategy Manual Research Group

継続　減薬　中止

今後の治療法を一緒に決めるための手引き

ADHD治療薬

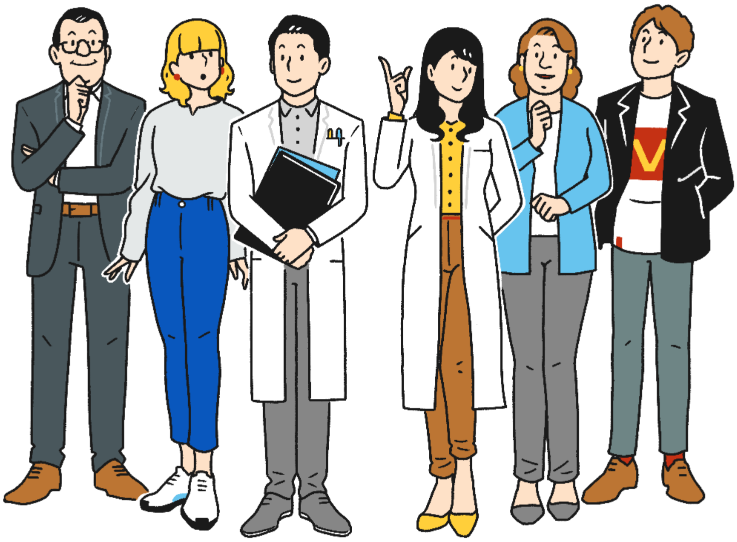

この手引きは**「向精神薬の出口戦略ガイドライン」**にもとづいて作成されています

もくじ

この手引きについて …… 1
この手引きの使い方 …… 2
注意欠如・多動症（ADHD）とは …… 3
これからの治療の選択肢を理解しましょう
　薬物療法について考えてみましょう …… 4
　薬物療法以外の治療を考えてみましょう …… 5
　これからの治療について考えましょう …… 6
　　選択肢1『ADHD治療薬の服用を 継続する』
　　選択肢2『ADHD治療薬を服用を 中止する』
各選択肢の長所・短所の例 …… 7
ADHD治療薬の服用を中止した結果について …… 8
自分にとって重要なこと …… 9
診察で話し合うための準備 …… 10
『ADHD治療薬の服用を 中止する』場合
　薬物療法以外の工夫を続けましょう …… 11
　薬物療法の中止方法 …… 12

付録1「よりよい日常生活を送るためにできること」 …… 14
付録2「症状チェックリスト」 …… 17
付録3「ADHD治療薬に関するよくある質問と回答」 …… 21
付録4「この冊子の子どもへの利用について」 …… 23

この手引きについて

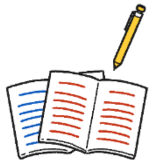

この手引きは、注意欠如・多動症（ADHD）治療薬を服用し、その症状が十分に軽減している状態が続いている方が、この先の治療を続けるかどうかについて意思決定をするために役立てられるように作成されています。

ADHD治療薬の服用を継続すること、中止することのいずれにも長所と短所があります。そのためどちらの選択肢が優れているとは限りません。

主治医は、これまでの治療経過やあなたの現在の状態について説明します。また、この手引きのなかでわからないことがあれば、主治医が教えてくれます。ですから、すべてをあなただけで決めなければいけないわけではありません。あなたの治療について、ご自身の気持ちを最大限に尊重し、これから今後の治療の方針を一緒に決めていきましょう。

【この手引きの対象になる方】

・ADHDと診断され、ADHD治療薬による治療を受け、その症状が改善した状態が持続している成人の方

【この手引きの対象とならない方】

・ADHDの症状が十分に改善していない方
・ADHDの症状が改善して、まだ間もない方
・ADHD以外の発達障害や、他の精神疾患を併存している方

ご自身がこの手引きの対象となるかわからない方は
医師に相談しましょう

この手引きの使い方

　この手引きは、医療者と話し合いながら、この先の治療法を選ぶためのものです。この手引きを自宅に持ち帰ってよく読み、ご家族などに相談するなどし、十分に検討しながら、今後の治療の方針を一緒に考えていきます。

手引きをよく読みます
○をつけたりメモ欄に
記入したりします

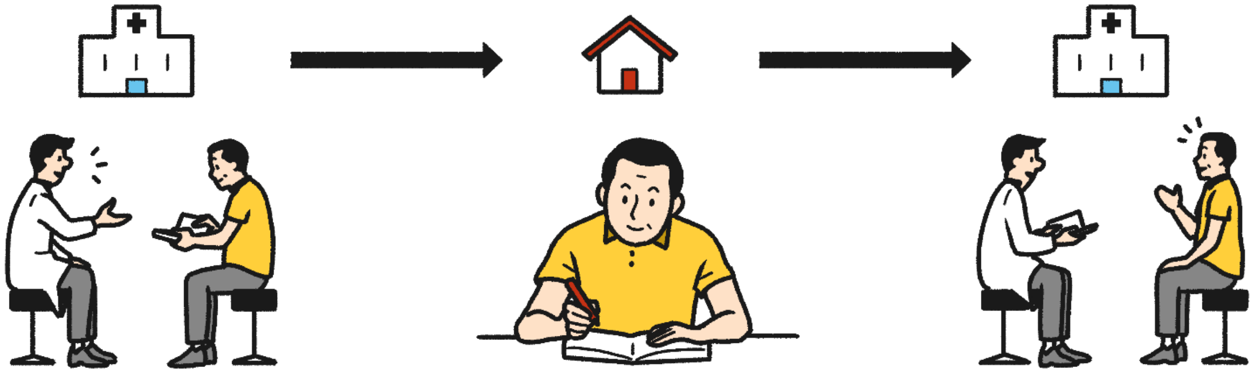

診察で

・現在の状態
・治療の選択肢
　について確認します

診察で

・○ をつけた項目や
　メモした内容について
　話し合います
・今後の方針を決めます

※一度で決まらない場合は
また持ち帰って検討します

注意欠如・多動症（ADHD）とは

注意欠如・多動症（attention-deficit/hyperactivity disorder: ADHD）は、12歳になる前から、学校、家庭、職場などの2つ以上の場面で同年代の方と比べて、注意を保ち続けることが難しかったり、落ち着きがなかったり、計画的な行動ができないために、社会生活・学業・日常生活に困難を来している状態です。

生活上の工夫や環境を整えることで、日々の暮らしにくさが楽になることがあります。それでも、つらさが続く場合には、薬物療法を併用することもあります。治療によって、日々の生活がしやすくなります。

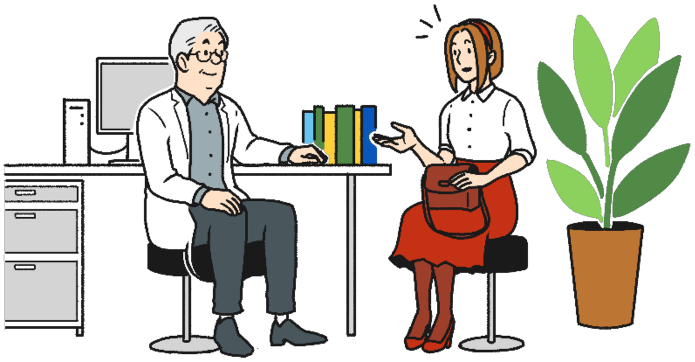

薬による治療により症状の改善が続いている場合の、この先の治療の選択肢をみていきます ☞

ステップ 1 これからの治療の選択肢を理解しましょう

薬物療法について考えてみましょう

ADHD治療薬は、ドパミンやノルアドレナリンといった神経伝達物質の働きを高め、ADHDがある人では働きが弱いとされる脳の活動を高めてくれます。

精神刺激薬

お薬：

メチルフェニデート徐放錠【コンサータ】
リスデキサンフェタミン【ビバンセ】*
*成人への適応未取得、18歳以前から服用の場合のみ継続可

特徴：

効果の持続は半日である
食欲低下、体重減少、不眠、血圧・心拍数増加がみられることがある
依存・乱用のリスクがあり、流通規制がしかれている**
**処方可能な医師、調剤可能な薬局が限られ、処方を受けるときには
カードなどを提示しなければならない

非精神刺激薬

お薬：

アトモキセチン【ストラテラ、アトモキセチン】
グアンファシン徐放製剤【インチュニブ】

特徴：

効果の持続は終日である
依存・乱用のリスクが低い
食欲低下、嘔気、血圧上昇、頻脈など
〔アトモキセチン〕液剤の選択肢がある、ジェネリック製剤がある
〔グアンファシン〕血圧低下、鎮静/眠気、頭痛など

ADHD治療薬に関するよくある質問 ➡ 巻末 付録3

薬物療法以外の治療を考えてみましょう

・**環境調整**

暮らしやすく、能力を発揮しやすくするために
環境を整えたり、周囲の理解を得る

・**心理教育**

ADHDの特性を知り、生活の工夫をこらす

・**行動療法的介入**

ADHDのある人が身につけたい行動を増やすために
かかわりかたを工夫する

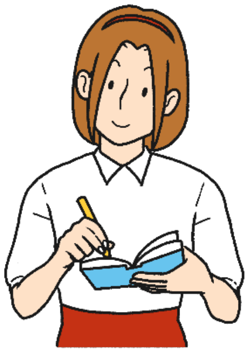

これからの治療について考えましょう

薬物療法以外のよりよい日常生活を送るためにできることは、これからの治療の選択にかかわらず継続していきます（付録1）

選択肢1　『ADHD治療薬の服用を継続する』

選択肢2　『ADHD治療薬の服用を中止する』

いずれの選択をおこなったとしても、その後の経過を定期的に評価して、治療を見直します（付録2）

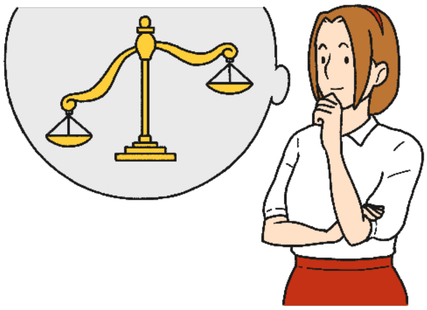

つぎはそれぞれの選択肢の長所・短所を比較してみましょう ☞

各選択肢の 長所・短所の例

ステップ 2 各選択肢の長所・短所について理解を深めます

薬物療法における一般的な長所・短所の例

	**選択肢1** ADHD治療薬の服用を **継続する**	**選択肢2** ADHD治療薬の服用を **中止する**
**長所** ☺	• ADHD症状を悪化させない<br>• 生活機能や生活の質を悪化させない	• 副作用がなくなる<br>• 経済的負担が減る<br>• 通院の負担が減る
**短所** ☹	• 副作用が持続する<br>• 現状の経済的負担が続く<br>• 現状の通院の負担が続く	• ADHD症状が悪化する可能性がある<br>• 生活機能や生活の質が悪化する可能性がある

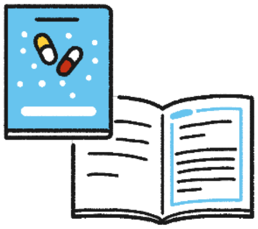

ADHD治療薬の服用を中止した結果について

ADHD治療薬で十分な症状の改善が持続した人が、それぞれの選択肢を選択した場合、ADHD症状の悪化がどの位の割合でみられるかを示しました[1)]。下の図は、100人中何人の割合で症状の悪化がみられるかを示しています。

選択肢1 ADHD治療薬の服用を **継続する**	選択肢2 ADHD治療薬の服用を **中止する**

ADHDと診断され、ADHD治療薬による治療を受け、
その症状が改善した状態が3週～52週間持続している成人の場合、

ADHD治療薬の服用を継続していても、その後4-25週の間にADHD症状が悪化する人が100人中5人います

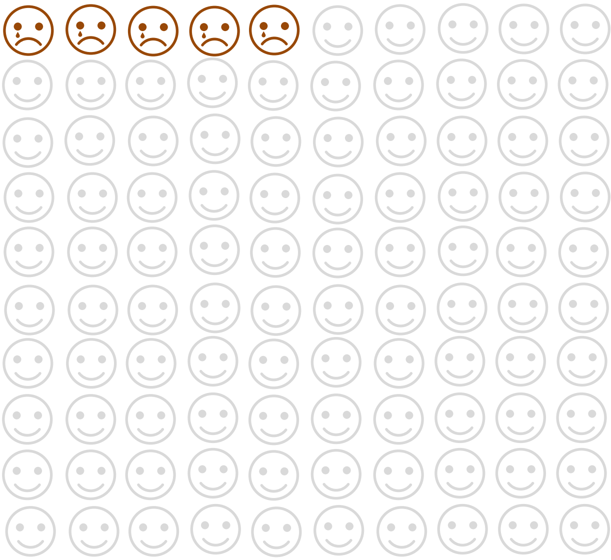

ADHD治療薬を中止したときその後4-25週の間にADHD症状が悪化する人が100人中22人います

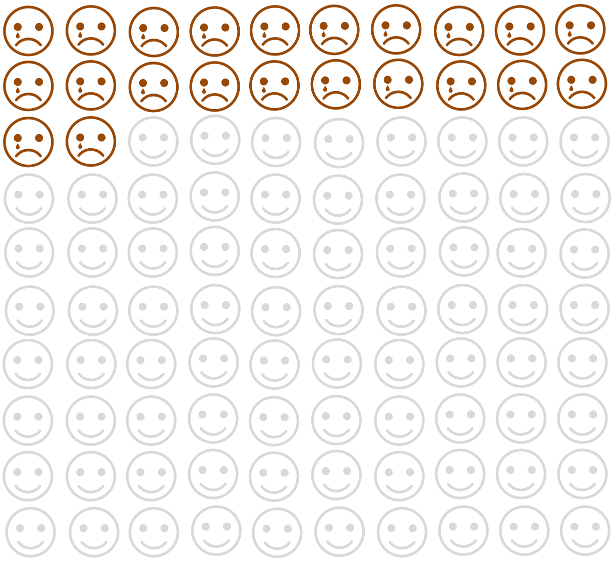

ADHD症状の悪化率には、統計的に意味のある違いがありました

自分にとって重要なこと

ステップ 3 あなたにとって重要なことを整理します

以下に、各選択肢を選ぶおもな理由をあげました。それぞれあなたにとってどのくらい重要ですか？あてはまる数字に○をつけ、重みづけをしてみましょう。

選択肢1『ADHD治療薬の服用を継続する』理由

	重要でない					重要である
（例）ADHD症状を悪化させない	0	1	2	3	4	5
（以下、自由に記載）						
・	0	1	2	3	4	5
・	0	1	2	3	4	5
・	0	1	2	3	4	5
・	0	1	2	3	4	5

選択肢2『ADHD治療薬の服用を中止する』理由

	重要でない					重要である
（例）副作用が消失する	0	1	2	3	4	5
（以下、自由に記載）						
・	0	1	2	3	4	5
・	0	1	2	3	4	5
・	0	1	2	3	4	5
・	0	1	2	3	4	5

診察で話し合うための準備

ステップ 4 医師との話し合いにむけた準備をします

あなたの考えをもとに、**選択肢1『ADHD治療薬の服用を継続する』選択肢2『 ADHD治療薬の服用を中止する』**について、診察のなかでご相談します。

疑問や気になったことなどいまの気持ちや考えを書いておきましょう

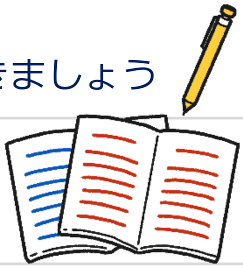

『ADHD治療薬の服用を中止する』場合

薬物療法以外の工夫を続けましょう

ステップ 1 これまで効果のあった日常生活の工夫をより意識して続けるようにします

環境調整

・あまりにも多くのやることを抱えていたり、優先順位をつけることが難しいことを抱えている場合には、ひとつずつ整理しておこなったり、メモなどで整理してから取り組むようにしましょう。

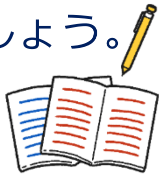

心理教育

・治療薬をやめることは、ADHDでなくなった、ということを意味するのではありません。これまで以上に、ご自身の得意、不得意に合わせて工夫した生活をしていきましょう。

行動療法的介入

・薬物療法を中止すると、ADHD症状が再び見えてきたりすることがあるかもしれません。しかし、本人は精一杯努力しているはずです。ご家族は否定的な言葉がけでなく、ご本人ができていることを認め、励ましてあげてください。

『ADHD治療薬の服用を中止する』場合

薬物療法の中止方法

ステップ 2 ご自身の処方薬にあわせた中止方法を選択します

治療薬1 『コンサータ/ビバンセ』

減量または徐々に休薬日を増やしながら、ADHD症状が悪化しないかみていきます。

治療薬2 『ストラテラ』

少しずつ減らさなくても中止することが可能ですが、不安が高まったり血圧などへの影響をさけるため、徐々に減量しながら、ADHD症状の悪化がないかみていきます。

治療薬3 『インチュニブ』

急な減量または中止により、血圧上昇及び頻脈があらわれることがあります。そのため、ゆっくりと減らしながら中止します。中止の方法については主治医と十分に相談してください。

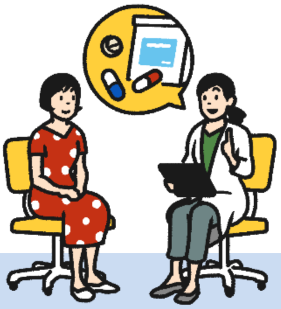

付録1

「よりよい日常生活を送るためにできること」

付録2

「症状チェックリスト」

付録3

「ADHD治療薬に関するよくある質問と回答」

付録4

「この冊子の子どもへの利用について」

付録1 よりよい日常生活を送るためにできること

一度にすべてを改善する必要はありません。
できそうなことから1 ～ 2個選んで、
一定期間取り組んでみましょう！

● 日常生活で、片付けが苦手な人

完璧を目指すと、目標がなかなか達成できず、だんだんつらくなってしまいます。まずは、完璧を目指さず、できることから手をつけてみましょう。

例えば、片づける物と場所が一致するよう、一度に持っておくものを減らしたり、わかりやすいように目印をつけて物の置き場所を決めたり、タイマーで1日に15分間と設定し物事に取り組む時間を区切って短い時間で自分に合った集中の仕方を見つけたりするなどの工夫もあります。

うまくいかないときは、終わったら○○しよう！と自分を励ますことや、どうしてもできないことは思い切って手放すことも一つです。イライラ対策に、運動や音楽鑑賞など、自分の好みに応じた心を落ち着ける取り組み（クールダウンの確保）が一つあるとよいでしょう。

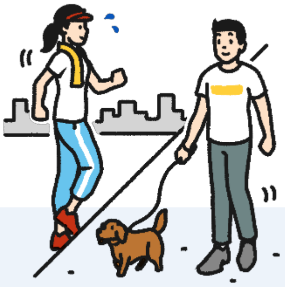

● 学校や仕事で、順序立てが苦手な人

複数の作業や仕事がある場合に、どの作業もこなそうと思うと、どれも中途半端になってしまいます。

まずは、作業を小分けにし、1つずつこなしてみましょう。優先順位を書き出し、優先順位の高い順にやるべきことをする（一覧表での順序立て）をしてみましょう。

うまくいかないときは、作業を小分けにしたあと、優先順位を書き出すことも有効でしょう。TO DO管理アプリなどで優先事項を決めて、大きな仕事をスモールステップに分解し、作業にとりかかるハードルを下げてみることも有効でしょう。

● 日常生活、学校や仕事で、忘れものの多い人

持ち物や予定を事前に確認しても、忘れ物がなくならないという人は、確認する機会を増やしましょう。

まずは、必要なものは玄関やドアの前などの日常生活の動線上に置いてみましょう。置き場所を決めるというのが基本ですが、鍵であればマグネットシートで鉄製のドアに貼りつけたり、キーチェーンを使ってかばんと一体化させたりする工夫ができます。

うまくいかないときは、仕事の始めと終わりに、状況を整理する時間をとること、職場からの持ち出しを減らすこと（職場に必要な筆記用具や手帳は置いてくるなど）、忘れたときの対策として身近な人に予備の書類を渡しておく。といったことも有効でしょう。

● **仕事や人間関係で、約束や期日を守れない人**

締め切りを守るためには、予定を具体化する方法が有効です。

まずは、スケジュールをわかりやすい表にし、人目につく所に貼ってみましょう。カレンダーアプリと連動したリマインダー機能を使ったり、スケジュール管理のアプリやソフトなどを使ってみましょう（ハイテク機器の活用）。

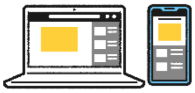

うまくいかないときは、スケジュールには自分で思っている以上の余裕をもたせることや、用事を安請け合いせず周囲に相談してから返事をすることも有効でしょう。

● **余計なひと言を言ってしまったり、かっとしてしまいがちな人、否定的な気持ちになりやすい人**

気になることがあったり、思いに沿わないことがあると、つい余計なひとことを言ってしまったり、気持ちを爆発させてしまうことがあります。また、罪悪感や恥ずかしさといった感情を感じるかもしれません。

このことが人間関係や仕事の妨げになっていると感じたら、まずは、気持ちを表現する前に一呼吸おくのがよいでしょう。休憩して一旦その場を離れることも一つです。また、日頃から自分の気持ちを押さえ込むだけでなく、怒りと上手に付き合うコツ（アンガーマネージメント）をみつけたり、気分転換をしてイライラを解消しておくことが大切です。

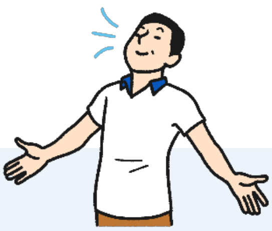

付録2 **症状チェックリスト**

以下のような評価項目があります。症状が悪化した場合には、心理社会的治療を強化するか、薬物療法を再開することも選択肢になります。

日常生活の評価

成人期ADHDの日常生活チェックリスト

Questionnaire **A**dult ADHD with **D**ifficulties（QAD）

QADは、毎日の生活のどのような場面でADHDの症状から生じる困難がみられるかを、ご本人･主治医が把握できる、成人のADHD患者の生活機能を評価するためのツールです。

成人のADHD患者が苦手とする、あるいは困難であると感じる場面を想定し、起床から就寝までの1日の流れに沿って生活機能を評価できる構成となっています。

ADHDの治療開始時から定期的にチェックを続けることで、生活上の困難がどの程度改善されたかも知ることができます。

機能障害の評価

成人自己評価式ワイス機能障害尺度

Weiss **F**unctional **I**mpairment **R**ating **S**cale **S**elf-Report（WFIRS-S）

WFIRS-Sは、成人のADHD患者が生活上のどの場面で困難を抱えているかを評価するためのツールです。

対象となる生活場面は、家族･職場･学校･生活上のスキル･自己概念･社交･リスクの7領域です。

ADHDの治療開始時から定期的にチェックを続けることで、生活上の困難がどの程度改善されたかも知ることができます。

成人期ADHDの日常生活チェックリスト[2,3)]

記入する日付、次回の診察日等を空欄に記入し、現在感じている症状を、1日の流れを思い出しながら4段階でチェックしてください。チェック後、次回診察時に主治医にご提示ください。

今日の日付	次回の診察日	用法	用量
/ /	/ /	1日 回	mg/日

0＝全く違う　1＝わずかにそう思う　2＝かなりそう思う　3＝全くその通り

		0	1	2	3
朝	1. 朝、速やかにベッドから起きられますか？	0	1	2	3
	2. 朝起きてから、速やかに身だしなみ（洗顔、歯磨き、着替えなど）を整えることができますか？	0	1	2	3
	3. 朝食を速やかに済ませることができますか？	0	1	2	3
	4. 朝から周囲とのトラブルや言い争いなどなく過ごせていますか？	0	1	2	3
日中	5. 仕事や家事を開始することが、スムーズにできますか？	0	1	2	3
	6. 周囲と同様に、計画的で段取りよく、集中して仕事や家事ができていますか？	0	1	2	3
	7. 周囲との対人関係はうまくいっていますか？	0	1	2	3
	8. 約束、用事や仕事を忘れずに覚えていることができますか？	0	1	2	3
	9. 大切なものをなくすことなく過ごせていますか？	0	1	2	3
	10. 必要なときに、ゆっくり待つことができますか？	0	1	2	3
	11. その日にやるべきことを最後まで達成できていますか？	0	1	2	3
	12. 余計なひと言や先走った行動がないように過ごすことができていますか？	0	1	2	3
	13. 落ち着きがないとかうるさいとか言われることなく過ごせていますか？	0	1	2	3
	14. 余暇活動に問題なく参加できていますか？	0	1	2	3
夜	15. 特定の事柄（パソコンや携帯電話、ゲーム、パチンコ、飲酒、喫煙など）に過度に没頭せず過ごせていますか？	0	1	2	3
	16. 段取りよく、待つことができていますか？	0	1	2	3
	17. 生活リズム（睡眠覚醒）は上手くいっていますか？	0	1	2	3
1日を通して	18. 気分の落ち込みや不安なく、自信を持って過ごせていますか？	0	1	2	3
	19. 混乱やトラブルなく、過ごせていますか？	0	1	2	3
	**チェック項目数×点数 を記入して下さい。→**	0			
	**合計点を算出して下さい。→**	合計 点			

成人自己評価式ワイス機能障害尺度[4)]

記入する日付、次回の診察日等を空欄に記入し、現在感じている症状を4段階でチェックしてください。<br>2点以上の項目については、機能障害があると考えられます。次回診察時に主治医にご提示ください。			
今日の日付	次回の診察日	用法	用量
/　　/	/　　/	1日　　　回	mg/日

0＝全くない　1＝ときどき　2＝しばしば　3＝いつも　□＝該当せず

		0	1	2	3	□
A 家族	1. 家族とトラブルになる	0	1	2	3	□
	2. 配偶者・パートナーとトラブルになる	0	1	2	3	□
	3. 自分のことをするのに家族に頼る	0	1	2	3	□
	4. 家族の喧嘩の原因をつくる	0	1	2	3	□
	5. 家族が一緒に楽しむのを妨げる	0	1	2	3	□
	6. 家族の面倒を見ることができない	0	1	2	3	□
	7. 自分のしたいことと家族のしたいこととのバランスを取ることができない	0	1	2	3	□
	8. 家族相手に自分をコントロールできなくなる	0	1	2	3	□
B 職場	1. 必要な責務を果たすことができない	0	1	2	3	□
	2. 効率的に仕事をこなすことができない	0	1	2	3	□
	3. 上司とトラブルになる	0	1	2	3	□
	4. 仕事が長続きしない	0	1	2	3	□
	5. 仕事を解雇される	0	1	2	3	□
	6. チームで働くことができない	0	1	2	3	□
	7. 欠勤する	0	1	2	3	□
	8. 遅刻する	0	1	2	3	□
	9. 新たな仕事に取りかかることができない	0	1	2	3	□
	10. 仕事で自分の潜在能力を出し切れない	0	1	2	3	□
	11. 業績を上司から低く評価される	0	1	2	3	□
C 学校	1. ノートをうまく取れない	0	1	2	3	□
	2. 課題をやり遂げられない	0	1	2	3	□
	3. 効率的に仕事をこなすことができない	0	1	2	3	□
	4. 教師とのトラブルがある	0	1	2	3	□
	5. 学校の管理者（学務課など）とのトラブルがある	0	1	2	3	□
	6. 学校の在籍に必要な最低限の単位を取ることが難しい	0	1	2	3	□
	7. 授業に欠席する	0	1	2	3	□
	8. 遅刻する	0	1	2	3	□
	9. 新しい課題に取りかかることができない	0	1	2	3	□
	10. 学業で自分の潜在能力を出し切れない	0	1	2	3	□
	11. 成績が安定しない	0	1	2	3	□

0＝全くない　1＝ときどき　2＝しばしば　3＝いつも　□＝該当せず

		0	1	2	3	□
D 生活上のスキル	1. 過度のあるいは不適切なほどインターネットやテレビゲームをするか、テレビを観る	0	1	2	3	□
	2. 程よく身なりを整えられない	0	1	2	3	□
	3. 外出するときの準備をすることができない	0	1	2	3	□
	4. 寝床につくのが難しい	0	1	2	3	□
	5. （栄養摂取が不十分など）食事摂取に関する問題がある	0	1	2	3	□
	6. 性交上の問題がある	0	1	2	3	□
	7. 睡眠の問題がある	0	1	2	3	□
	8. けがをしやすい	0	1	2	3	□
	9. 運動を避ける	0	1	2	3	□
	10. 医者・歯医者の予約を守ることができない	0	1	2	3	□
	11. 家庭内のこまごましたことをこなすことができない	0	1	2	3	□
	12. 金銭管理の問題がある	0	1	2	3	□
E 自己概念	1. 自己嫌悪に陥っている	0	1	2	3	□
	2. 自分自身に対する苛立ちが溜まっている	0	1	2	3	□
	3. 落胆した状態である	0	1	2	3	□
	4. 自分の人生は幸せだと思わない	0	1	2	3	□
	5. 自分は能力に欠けると感じている	0	1	2	3	□
F 社交	1. 口論になる	0	1	2	3	□
	2. 上手く他人と協力できない	0	1	2	3	□
	3. 他人と上手くやっていけない	0	1	2	3	□
	4. 他人と一緒に楽しめない	0	1	2	3	□
	5. 趣味活動に参加することができない	0	1	2	3	□
	6. 友人を作ることができない	0	1	2	3	□
	7. 友人関係を保つことができない	0	1	2	3	□
	8. 不適切な発言をする	0	1	2	3	□
	9. 近所から苦情をいわれる	0	1	2	3	□
G リスク	1. 乱暴な運転をする	0	1	2	3	□
	2. 運転しながら他のことをする	0	1	2	3	□
	3. 運転中に（割り込まれたりすると）突然キレてしまう	0	1	2	3	□
	4. ものを壊す、傷つける	0	1	2	3	□
	5. 法に触れることをする	0	1	2	3	□
	6. 警察沙汰になる	0	1	2	3	□
	7. 喫煙する	0	1	2	3	□
	8. マリファナを吸う	0	1	2	3	□
	9. 飲酒する	0	1	2	3	□
	10. “ストリートドラッグ”を使用する	0	1	2	3	□
	11. 避妊（出産計画、コンドーム）なしで性交する	0	1	2	3	□
	12. 性的に不適切な行為をする	0	1	2	3	□
	13. 身体的な暴力を振るう、小突く	0	1	2	3	□
	14. 言葉の暴力をふるう	0	1	2	3	□

付録3 ADHD治療薬に関するよくある質問と回答

● 一生飲み続けないといけないのですか？

　現時点では、どの程度長期にADHD治療薬を飲み続けないといけないのか、明確な基準や推奨はありません。ADHD治療薬によるADHD症状の改善には、長期的な薬物の継続が望ましいと考える専門家もいます。しかし、その症状が十分に軽減している状態が続いている方には、ADHD治療薬を中止してみることも1つの選択肢となります。主治医と相談してください。

● 徐々に効果が弱くなり量が増えるのでは？

　医師の指示通りに内服している場合には、段々と効果が弱くなり量が増えることはないと考えられています。就職や結婚など、社会的な負担が増えるといった環境の変化によって、ADHD症状による困難が大きくなったように感じることがあります。そのような場合でも、治療薬の効果が弱くなったわけではありません。まずは適切な心理社会的治療や環境調整について主治医と相談してください。

● この冊子を子どもにも利用することができますか？

　付録4をご覧下さい。

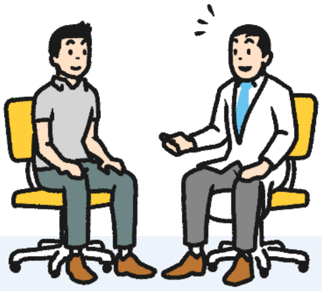

- **子どもを欲しいと思っていますが、薬を飲み続けて良いでしょうか？**

　添付文書では、インチュニブは妊婦又は妊娠している可能性のある婦人には投与しないこととされています。コンサータは、妊婦又は妊娠している可能性のある婦人には投与しないことが望ましいとされています。ストラテラとビバンセは、治療上の有益性が危険性を上回ると判断される場合にのみ投与することと記載されています。妊娠がわかったら、主治医や薬剤師と相談しましょう。

- **服薬中に授乳しても良いでしょうか？**

　ADHD治療薬は母乳への移行があることが知られています。添付文書では、ADHD治療薬を内服中は授乳を避けることが記載されています。授乳を続けるためには、ADHD治療薬を中止することも選択肢になりますので、主治医や薬剤師と相談しましょう。

- **その他（医師への質問をメモしておきましょう）**

  -
  -
  -
  -

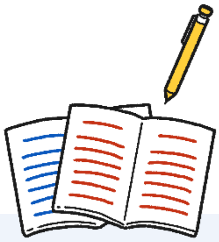

付録4 **この冊子の子どもへの利用について**

子どもの場合にも、この冊子で書かれたことの多くが当てはまりますが、いくつかの相違があります。子どもは発達の過程にありますので、薬物療法、特に精神刺激薬で生じる食欲低下、体重減少（期待される体重増加が見られない）、成長抑制（身長の伸びが遅くなる）といったことが懸念されます。実際には、大人になったときの身長に与える影響は極めて小さいと考えられています。しかし、体重や身長の伸びについて経過を見ること、特に食欲が低下している場合、間食で補ったり、休薬日をもうけることも考慮されます。

ADHD治療薬で一定期間にわたり十分な症状の改善をみとめた子どもが、治療薬の服用をやめた場合、ADHD症状の悪化がどの位の割合でみられるかを示しました（次のページを参照）。

薬物療法を継続するか、中止するか、について、それぞれの選択肢の長所、短所を考え、子どもにとっての重要度を考えて治療の決定を行うことができます。しかし、最も重要なことは、このような意思決定を誰がどのように行うのか、ということです。

治療上の意思決定を行うためには、それぞれの選択肢について理解し、その選択を行ったときの結果を予測すること、自分の気持ちで自由に選択していいことを理解する必要があります。しかし、子どもには難しいことがあります。そのために、子どもにとって最善の結果となるように保護者が代諾するわけです。

選択肢1 ADHD治療薬の服用を **継続する**	選択肢2 ADHD治療薬の服用を **中止する**

ADHDと診断され、ADHD治療薬による治療を受け、
その症状が改善した状態が6週～52週間持続している**子ども**の場合、

ADHD治療薬の服用を継続していても、その後6-36週の間にADHD症状が悪化する人が100人中26人います

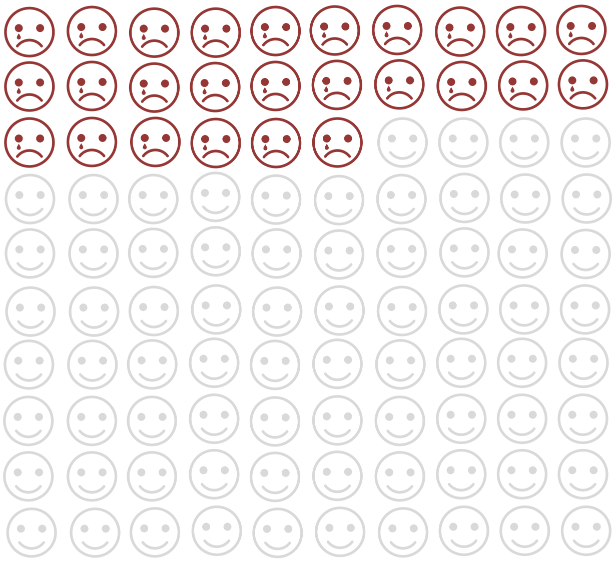

ADHD治療薬を中止したとき その後6-36週の間にADHD症状が悪化する人が100人中48人います

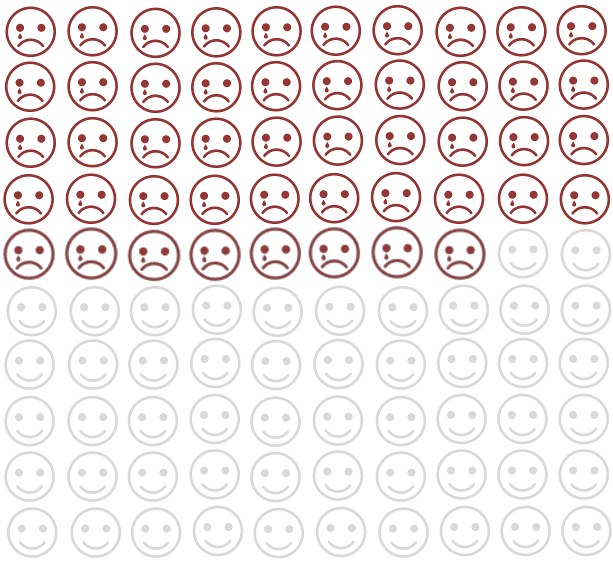

ADHD症状の悪化率には、統計的に意味のある違いがありました

一方、子どもには、その子の能力からみてわかる言葉でできる限り説明を受け、治療の選択肢に賛意（アセント）を表明することも大切です。アセントに必要な力は、子どもの年齢や知的能力によって大きく異なるので、この冊子をそのまま使用できるわけではありません。主治医の先生との対話に、より多くの時間をかけて、保護者も子どもも納得のいく治療選択をしましょう。

おわりに

●自分にあった治療法を選ぶために

　治療の選択肢には、それぞれ長所と短所があります。この手引きは、それらをよく理解し、自分にとって重要なことを明らかにしながら医療者と話し合い、あなたに合った選択ができるよう作られています。

●手引きの開発プロセス

　この手引きに掲載した情報は、ADHDの治療を経験されたことのある方々の声や意見を反映させました。精神科の専門家のチェックも受けています。

また、この手引きは厚生労働科学研究費補助金・障害者政策総合研究事業「平成29年度～30年度：向精神薬の処方実態の解明と適正処方を実践するための薬物療法ガイドラインに関する研究（H29-精神-一般-001）」および「平成31年/令和1年度～2年度：向精神薬の適切な継続･減量･中止等の精神科薬物療法の出口戦略の実践に資する研究（19GC1012）」により作成しました。特定の企業からの援助は受けていません。

●手引きの更新

　この手引きは、必要に応じて見直しと更新をおこないます。

※ ここに掲載された情報は、医療者と話し合いながら対処法や治療法を決める際の手引きとなるものであり、医療者のアドバイスの代わりになるものではありません。

文献

1) Tsujii, N., Okada, T., Usami, M., Kuwabara, H., Fujita, J., Negoro, H., Kawamura, M., Iida, J., Saito, T., 2020. Effect of Continuing and Discontinuing Medications on Quality of Life After Symptomatic Remission in Attention-Deficit/Hyperactivity Disorder. The Journal of Clinical Psychiatry 81(3): 19r13015.

2) 市川宏伸、今村明、根來秀樹（監修）. 成人期ADHDの日常生活チェックリスト. https://adhd.co.jp/pdf/Adult_QAD_checksheet.pdf

3) 井上清子. 2019. 成人期ADHD日常生活チェックリスト（QAD）の信頼性と妥当性についての一考察. 生活科学研究 = Bulletin of Living Science, 9-16.

4) Takeda, T., Tsuji, Y., Kanazawa, J., Sakai, T., Weiss, M.D., 2017. Psychometric properties of the Japanese version of the Weiss Functional Impairment Rating Scale: Self-Report. Attention deficit and hyperactivity disorders 9, 169-177.

作成・向精神薬出口戦略マニュアル研究班・ADHD治療薬グループ

作成：2021年3月　　更新予定日：2023年3月

この手引きは「ADHD治療薬の適正使用・休薬ガイドライン」および「向精神薬の出口戦略ガイドライン」にもとづいて作成されています

©2021,向精神薬出口戦略マニュアル研究班
無断複写・無断転載はご遠慮ください

Supplementary material 3

International Patient Decision Aid Standards criteria met by current decision aid (Joseph-Williams et al., 2014)

**Item**	**1. Qualifying criteria**	**2. Certification Criteria**	**3. Quality Criteria**
Information	Describes the health condition or problem for which decision is required†<br>Explicitly states decision that needs to be considered†<br>Describes the options available for the index decision†<br>Describes positive features of each option†<br>Describes negative features of each option†	Shows the negative and positive features of options with equal detail†	Describes the natural course of the health condition or problem if no action is taken†<br>Makes it possible to compare the positive and negative features of available options†
Probabilities			Provides information about outcome probabilities associated with the options†<br>Specifies the defined group of patients for whom the outcome probabilities apply†<br>Specifies the event rates for outcome probabilities†<br>Allows the user to compare outcome probabilities across options using the same time period †<br>Allows the user to compare outcome probabilities across the same denominator†<br>Provides more than 1 way of viewing the probabilities (eg, words, numbers, diagrams)†

Values	Describes what it is like to experience consequence of the options†		Asks patients to think about which positive and negative features of options matter most to them†
Guidance			Provides a step-by-step way to make a decision†<br>Includes tools like worksheets or lists of questions to use when discussing options with a practitioner†
Development			Development process included a needs assessment with clients or patients†<br>Development process included a needs assessment with health professionals†<br>Development process included review by clients/ patients not involved in producing the decision support intervention†<br>Development process included review by professionals not involved in producing the decision support intervention†<br>Field tested with patients who were facing the decision‡<br>Field tested with practitioners who counsel patients who face the decision‡
Evidence		Provides citations to the evidence selected†<br>Provides a production or publication date†<br>Provides information about the update policy†<br>Provides information about the levels of	Describes how research evidence was selected or synthesized†<br>Describes the quality of the research evidence used†

		uncertainty around the event or outcome probabilities†	
Disclosure		Provides information about the funding source used for development†	Includes authors'/developers' credentials or qualifications†
Plain Language			Reports readability levels†
Evaluation		Describes what the test is designed to measure‡	Evidence improved match between preferences of the informed patient and the option chosen‡<br>Evidence patient decision aid helps patients improve their knowledge about options' features‡
† Criteria met by the developed decision aid<br>‡ Criteria to be met with effectiveness testing, not applicable for the current decision aid			

Supporting Information 4

A decision aid for attention-deficit/hyperactivity disorder (ADHD)
Considering discontinuation of medications

—Doctor's manual—

【Guidance for making decisions with your healthcare professionals】

It is important to have shared decision making (SDM) in which doctors evaluate the patients' conditions and present the best possible options while making decisions with the patients rather than merely obtaining their consent. This decision aid (DA) booklet for ADHD is the first step in the process of performing SDM, and it shows the advantages and disadvantages of future treatment based on the current condition of the patient. It is designed to avoid bias, confirm the patient's preferences and intentions, and to note any questions that arise in the process hat might lead to a useful discussion between the doctors and patients. **It is worth noting that the final decision is made by the doctor and the patient together in a two-way interaction at the next step of the DA rather than giving the patient the DA to make the decision alone.**

The DA uses SDM to determine whether to continue or discontinue ADHD medications for adults who have been taking at least one ADHD medication and have experienced remission with the treatment. It is a tool for making decisions along with patients. This physician's manual contains guidelines for the proper use of DA.

【Eligibility for the guide】

The target audiences of this DA are those who have been taking ADHD medications and have experienced remission with the treatment. This guide provides data on relapse/recurrence with and without ADHD medications with an estimated remission period of 3–52 weeks (6–52 weeks for children). However, there is no clear standard in clinical settings although remission is maintained on a semi-annual or yearly basis. The perception of remission is shared by both doctors and patients, and it is often considered when the psychosocial situation surrounding the patient is stable. Healthcare professionals can use the first page of the booklet to discuss the current situation with the patient, confirm that the patient is in remission, and that they are in the process of discussing whether to continue or discontinue ADHD medications. This marks the starting point for SDM.

This booklet lists the following criteria:

<Those who are eligible for this guide>

Adults who have been taking at least one ADHD medication and have attained remission with the treatment.

<Those who are not eligible for this guide>

Individuals who failed to respond to ADHD medications and did not attain remission with those medications or attained remission in less than 3 months and had any other neurodevelopmental or psychiatric disorders.

【How to use this DA】

Use the second page to clarify how to use this guide. At this stage, patients are expected to know that they are respected. Do not make them feel uncomfortable because they may feel abandoned or sidelined from the decision making regarding treatment choices. On this page, the patients will be able to know that the guide is aimed at centering their feelings "while discussing with their healthcare professional" for selecting future treatment methods, including thinking "together" for future treatment policies. The DA explains how patients will be able to put a checkmark and write things down as they read, and how they can discuss their questions, impressions, and what they wrote down at the next visit.

Further treatment options

An overview of the goals of ADHD treatment (Page 3)

Before discussing future treatment options, page 3 provides an overview and demystifies the goals of ADHD treatment. ADHD medication is given when environmental conditioning or psychosocial treatment does not improve ADHD symptoms and when social dysfunction persists. It should be noted that even if ADHD medication is discontinued, psychosocial efforts may persists, so it is important to track the history of treatment.

Step 1 (Pages 4–6)

There are two options: pharmacological and nonpharmacological treatments of ADHD. Because the DA is intended for patients who have already experienced remission with the current medication, it does not mention what kind of effect it has or how strong or weak it is. It focuses on the patient's burden of duration of effects, side effects, distribution restrictions, etc., which are required to decide whether to continue or discontinue the ADHD medication. Whether it is the effects or side

effects of ADHD treatments or psychosocial efforts, the content varies considerably from patient to patient. A thorough review of these pages will generate important information for subsequent treatment decisions.

On page 6, you will see two options: "Continuing ADHD medications" and "Discontinuing ADHD medications." It does not mean that either is correct. Moreover, the choices you have just made do not limit your lifelong treatments; however, you should review the use of medications regularly and stop taking them although you may resume to take them later or take the medications even if you are required to stop taking them. This page explains that it is possible to resume medications and reduce patients' anxiety.

Step 2（Pages 7–8）

Here, we ask the patients to confirm the strengths and weaknesses of each option. However, looking at the available option, the division is not exclusive. The pages indicate that even if patients continue to take ADHD medications, the demands in their daily life may become excessive and symptoms may become apparent, thereby hindering their daily activities. Regarding side effects, some medications have side effects, whereas others do not. In some cases, patients may be overwhelmed with financial burden. Discontinuing medication may not change the frequency of hospital visits. Instead, it may require intensive psychosocial treatment. In some instances, stopping medications may reduce side effects and improve quality of life. It is just an example of general strengths and weaknesses, and it is important to consult with the patient based on this general description.

Page 8 shows the outcomes of the two options for adults with ADHD who were treated with ADHD medications and experienced remission for 3–52 weeks by either continuing or discontinuing medication. It shows the estimated percentage of ADHD symptom relapse among patients who have experienced remission as 5% and 22% for those continuing and discontinuing ADHD treatment, respectively, within 4–25 weeks (Reference 1). It is easy for patients to understand that stopping ADHD medications may worsen symptoms, but it is unimaginable that the symptoms may worsen even if the medication is continued. Meanwhile, whether 5 or 22 out of 100 people are big or small depends on their personal values. This result was based on a meta-analysis of previous reports although the result of examinations by many people has indicated a statistically significant difference. Regardless of the statistically significant difference, the expectations of individual patients are paramount. It is thus important to visually present the results as objective data so that the patient can appreciate them.

Step 3 (Page 9)

This page requires patients to assess the strengths and weaknesses of each of the options mentioned in step 2. The strengths and weaknesses of each patient are different, so these should be written freely by them. It asks them to rate their expectations from not important (0) to important (5) with the following instruction: during the examination, we will ask you about the details of each item that you have freely stated and use it as the basis for SDM.

Step 4 (Page 10)

This page requires patients to fill in any questions or concerns about the two options. It enables them to discuss whether to continue or discontinue the ADHD treatment based on the contents of steps 3 and 4. If the patients decide to discontinue ADHD medication, they will proceed to the next section to discuss how to continue psychosocial treatment and discontinue medications.

(If discontinuing medications) Further treatment options

Step 1 (Page 11)

See page 5 for psychosocial interventions other than ADHD medical treatments. It covers how you will continue those efforts after discontinuing ADHD medications, and how doctors and patients will work together following the discontinuation of ADHD medications.

Step 2 (Page 12)

It is likely that some patients may stop their medications suddenly following their decision to discontinue. Given the likelihood of relapse of symptoms at the point of discontinuation, it is better to emphasize gradual management of weight loss while taking into consideration the quality of life and physical health.

Appendices

The appendices describe specific psychosocial interventions (Appendix 1). In addition, they highlight the need for the continuous revision of the policy irrespective of the adopted options. They also emphasize the importance of evaluating ADHD symptoms at the time of taking the decision. Here, we have listed the Questionnaire Adult ADHD with Difficulties and the Weiss Functional Impairment Rating Scale Self-Report (Reference 4). In addition, Appendix 3 lists frequently asked questions and answers regarding ADHD medications, so please use them as needed. Appendix 4 describes the use of

this booklet for children. The procedure for making decisions about whether to continue or discontinue ADHD medications may be similar for children and adults. However, there are differences in the most important aspects of decision making, such as regarding who makes these decisions and how.

To make therapeutic decisions, healthcare providers need to understand each option, anticipate the consequences of making a choice, and understand that the patients are free to make their own choices. This can, however, be difficult for children. This DA informs parents to give their consent for the best results for their children. However, it is also important to simplify the wording of the treatment options as much as possible so that it can be understood by children and they can express their support (consent) for the treatment options. The ability required for consent varies greatly depending on the child's age and intellectual ability, so this booklet cannot be used specifically for children.

Ideally, children should be free to express their will even in front of their parents, and parents should respect their children's interests fully so as to make decisions based on their children's will. However, this is not always the case, especially in cases of abuse. Children are not always free to express their will, which makes the pros and cons of the parent's decision to a child more problematic. Therefore, doctors need to fully assess the environment in which children with ADHD are living before making a shared decision.

文献

1) Tsujii, N., Okada, T., Usami, M., Kuwabara, H., Fujita, J., Negoro, H., et al. (2020). Effect of continuing and discontinuing medications on quality of life after symptomatic remission in attention-deficit/hyperactivity disorder: a systematic review and meta-analysis. *The Journal of Clinical Psychiatry, 81*, 11514.

2) Questionnaire Adult ADHD with Difficulties (QAD)
https://adhd.co.jp/pdf/Adult_QAD_checksheet.pdf

3) Inoue, K. (2019). A study on the reliability and validity of Questionnaire Adult ADHD with Difficulties (QAD). *Bulletin of Living Science, 41*, 9-16.

4) Takeda, T., Tsuji, Y., Kanazawa, J., Sakai, T., & Weiss, M. D. (2017). Psychometric properties of the Japanese version of the Weiss Functional Impairment Rating Scale: self-report. *Attention Deficit Hyperactivity Disorders, 9*, 169-177.

注意欠如・多動症（ADHD）治療薬を
継続する/中止する
一緒に決めるための手引き
―医師用マニュアル―

【医療者と一緒に決定するための手引きについて】

医師が患者の状態を評価して最善と考える選択肢を呈示し、患者に同意を得るというのではなく、患者と一緒に意思決定を行うという共同意思決定（Shared Decision Making: SDM)が重要であると認識されています。この「医療者と一緒に決定するための手引き（Decision Aid: DA)」は、SDM を行うプロセスの第一段階となる資材で、現在の患者の状態を踏まえ、これからの治療の利点と欠点を偏りなくなく提示し、患者の好みや価値観を含めた意向を確認しながら、その過程で生じた疑問を書き記し、医師と患者の話し合う機会へとつなげていくために作成されています。特筆すべきことは、**患者に DA を渡して単独で決めさせるのではなく、最終的な意思決定は、DA の次のステップで医師と患者が双方向的なやりとりのなかで一緒に決めていく**ということです。

別紙の DA は、注意欠如・多動症（ADHD）の薬物療法によって、症状が十分に軽減し、寛解状態が維持されている患者について、薬物療法を継続するか/中止するかを、SDM の手法で患者と一緒に話し合いながら決めるためのツールです。この医師用マニュアルはこの DA を適切に使うための指針が記載されています。

【この手引きの対象となる方】

この手引きの対象が、ADHD 治療薬を服用し、その症状が十分に軽減している状態が続いている方です。この手引きのなかでは、ADHD 治療薬を継続した場合とやめた場合の再燃・再発のデータについて示していますが、そこでは寛解期間は 3～52 週（子どもの場合には 6～52 週）とされています。しかし、現実的には明確な基準はありませんが、半年や年の単位で寛解が維持されている場合となるでしょう。寛解しているという認識が医師、患者ともに共有されており、患者さんを取り巻く心理社会的な状況が安定している場合に検討されると思います。冊子の 1 ページ目を用いながら、現状について患者とともに話し合い、医師と患者が寛解状態にあることを確認し、治療薬の服用を継続するか/中止するかを話し合う段階にあることを確認し合うことが、SDM の出発点です。

この冊子では、以下のような基準を挙げています。

<この手引きの対象になる方>

・ADHD と診断され、ADHD 治療薬による治療を受け、その症状が改善した状態が持続している成人の方

<この手引きの対象とならない方>

・ADHD の症状が十分に改善していない方
・ADHD の症状が改善して、まだ間がない方
・ADHD 以外の発達障害や、他の精神疾患を併存している方

【この手引きの使い方】

2 ページ目を用いて、この手引きの使い方について明確にしましょう。ここまでの段階で、患者にはご自身の意思を尊重することは伝わっていると思いますが、治療選択に対する意思決定を丸投げされた

と感じさせてしまったり、そのことによって不安な気持ちにさせてしまうのでは、本末転倒です。この手引きは「医療者と話し合いながら」あなたの気持ちを最大限に活かし、これからの治療法を選択するためのものであることや、今後の治療の方針を「一緒に」考えていくためのきっかけであることを強調する必要があります。そのためには、自宅に持ち帰ってよく読み、ご家族などに相談するなどして、自分の考えや疑問を冊子に書き込んでくることをお願いします。

これからの治療の選択肢

ADHD の概要と治療の位置づけ（3 ページ）

これからの治療の選択肢について扱う前に、3 ページでは ADHD の概要と、治療の位置づけを振り返ります。ADHD の薬物療法は、環境調整や心理社会的な治療によって ADHD 症状が改善せず、社会的な機能障害が持続する場合に行われます。そして、薬物療法が中止されることがあっても、心理社会的な取り組みは継続されるわけですので、ここで、これまでの治療の歩みを振り返っておくことが大切です。

ステップ 1（4－6 ページ）

ADHD の薬物療法、薬物療法以外の治療について振り返ります。すでに現在の投薬で寛解を達成した患者を対象にしていますので、どのような効果があるのか、効果の強弱はどうかといったことは触れていません。治療薬の継続か中止かを決めるのに必要な、効果の持続時間や副作用、流通規制などに伴う患者の負担に焦点を当てています。薬物療法の効果や副作用、心理社会的な取り組みにせよ、患者によってかなり内容に違いがあります。このページを用いて十分に振り返ることが、その後の治療の意思決定にも大切な情報になります。

6 ページでは、『ADHD 治療薬の服用を継続する』と『ADHD 治療薬の服用を中止する』という二つの選択肢を確認します。いずれが正しいと言うことではありません。また、いま決定した選択が生涯にわたる治療を制約するのではなく、定期的な見直しをおこない、いったん服用を継続したとしても服用を中止したり、服用を中止していても ADHD 治療薬の服用を再開することも可能であることを説明し、患者の不安を軽減しましょう。

ステップ 2（7～8 ページ）

ここでは患者に各選択肢の長所・短所を確認していただきます。ただ、個々の選択肢をみると、このように単純に割り切れません。治療薬を継続していても、日常生活における要請が過剰になれば症状が顕在化したり、日常生活の支障も大きくなることがあります。副作用についても、副作用のある方、ない方があります。経済的負担についても、自己負担がない場合もあります。薬物療法を中止しても、通院の頻度が変わらなかったり、逆に濃密な心理社会的治療を要することもあるかもしれません。薬剤の注視により副作用が軽減し、生活の質が改善することもあります。あくまでも「一般的な長所・短所の例」であり、この一般的な記載をもとに患者と相談することが大切です。

8 ページには、ADHD と診断され、ADHD 治療薬による治療を受け、その症状が改善した状態が 3 週～52 週間持続している成人が、薬物療法を継続した場合と、中止した場合、どの程度の割合で症状の悪化がみられるかを示しています（文献 1)。治療薬の服用を中止して、症状が悪化する可能性がある、ということは患者にとっても理解しやすいですが、治療薬の服用を継続しても悪化することがある、ということは想像の範囲外かもしれません。100 人中 5 人と 100 人中 22 人が大きいか小さいかというこ

とは、個人価値観によっても判断は違ってきます。これは 100 人で調べた結果ではなく、既報のメタ解析ですので、たくさんの人で調べた結果で、統計学的にも有意差が得られています。しかし、統計学的な有意差があったからといって、個々の患者にとってどうなのか、そのことが患者の思いとしてどうなのかは別の問題です。客観性のあるデータとして視覚的に呈示し、患者に感じ取っていただくのがよいでしょう。

ステップ 3（9 ページ）

ステップ 2 で取り上げた各選択肢の長所・短所について、患者にとっての重要度を評価してもらいます。長所、短所は患者それぞれですから、あえて自由記載としています。その重要度を、重要でない（0）から重要である（5）の段階で評価してもらってください。診察のなかでは、個々の自由記載した項目について詳細を聞き、共同意思決定の材料にします。

ステップ 4（10 ページ）

2 つの選択肢に関する疑問や心配なことを患者に記入してもらいます。ステップ 3、4 の記載内容に基づき、ADHD 治療薬を継続するか/中止するかを話し合います。中止する方針となった場合は、次のセクションに進み、心理社会的治療の継続や薬物療法の中止方法について話し合います。

『ADHD 治療薬の服用をやめてみる』場合の薬物療法以外の工夫、薬物療法の中止方法

ステップ 1（11 ページ）

薬物療法以外の心理社会的な取り組みについては、5 ページでも確認しています。薬物療法を中止後もそれらの取り組みを継続していくことについて確認し、医師と患者が共同して、薬物療法中止後の生活に取り組むことを確認します。

ステップ 2（12 ページ）

患者のなかには、薬物療法の中止の方針を決定すると、即時中断してしまう人がいます。中止時の反跳症状もありますが、それだけではなく、徐々に症状だけでなく、生活面、身体面も視野に入れながら減量に取り組んでいくことを強調しましょう。

付録

付録には、具体的な心理社会的取り組みについて書いてあります（付録 1）。また、薬物療法を継続するにせよ、やめてみるにせよ、継続的な方針に見直しは行うべきであり、その際には症状評価をおこなうことが大切です。ここでは、成人期 ADHD の日常生活チェックリスト（文献 2，3）と成人自己評価式ワイス機能障害尺度（文献 4）をあげました。また、付録 3 には、薬物療法によくある質問と回答を挙げましたので、必要に応じてご活用ください。

付録 4 は、この冊子の子どもへの利用について書いてあります。薬物療法の継続か中止かをめぐって意思決定をする手順は、子どもも大人と概ね同様に進められるかも知れません。しかし、最も重要なことは、このような意思決定を誰がどのように行うのか、という意思決定の最も重要な点に違いがあるのです。

治療上の意思決定を行うためには、それぞれの選択肢について理解し、その選択を行ったときの結果を予測すること、自分の気持ちで自由に選択していいことを理解する必要があります。しかし、子ども

には難しいことがあります。そのために、子どもにとって最善の結果となるように保護者が代諾をするわけです。一方、子どもには、その子の能力からみてわかる言葉でできる限り説明を受け、治療の選択肢に賛意（アセント）を表明することも大切です。アセントに必要な力は、子どもの年齢や知的能力によって大きく異なるので、この冊子をそのまま使用できるわけではありません。

理想的な場合には、子どもは親の前でも自分の意思を自由に表現し、親は子の利益を最大限に尊重し、子どもの意思をくみ取りながら意思決定すると考えられます。しかし、そのような理想的な場合ばかりではありません。特に、虐待のある場合には、子は自らの意思を自由に表現できませんし、子の治療の意思決定を親が行うことの是非こそ問題になるのです。ですから、医師は、患者である子どもがどのような環境にいるかどうかを十分に評価してから共同意思決定を行う必要があります。

文献

1） Tsujii, N., Okada, T., Usami, M., Kuwabara, H., Fujita, J., Negoro, H., Kawamura, M., Iida, J., Saito, T., 2020. Effect of Continuing and Discontinuing Medications on Quality of Life After Symptomatic Remission in Attention-Deficit/Hyperactivity Disorder. The Journal of Clinical Psychiatry 81(3):19r13015

2） 市川宏伸、今村明、根來秀樹（監修）. 成人期 ADHD の日常生活チェックリスト. https://adhd.co.jp/pdf/Adult_QAD_checksheet.pdf

3） 井上清子. 2019. 成人期 ADHD 日常生活チェックリスト（QAD）の信頼性と妥当性についての一考察. 生活科学研究 = Bulletin of Living Science, 9-16.

4） Takeda, T., Tsuji, Y., Kanazawa, J., Sakai, T., Weiss, M.D., 2017. Psychometric properties of the Japanese version of the Weiss Functional Impairment Rating Scale: Self-Report. Attention deficit and hyperactivity disorders 9, 169-177.
